# Precision sniper for solid tumors: CAR-NK cell therapy

**DOI:** 10.1007/s00262-025-04106-z

**Published:** 2025-07-24

**Authors:** Sisi Li, Jiajie Jing, Yueming Chen, Enjie Chi, Bingyan Wang, Ziwen Xie, Wenya Yang, Hongqiang Shen, Jianping Pan

**Affiliations:** 1 Department of Clinical Medicine, Hangzhou City University School of Medicine, 51 Huzhou Street, Hangzhou, 310015 People’s Republic of China; 2 Institute of Translational Medicine, Hangzhou City University, 51 Huzhou Street, Hangzhou, 310015 People’s Republic of China; 3 Department of Clinical Laboratory, Children’s Hospital, Zhejiang University School of Medicine, National Clinical Research Center for Child Health, Hangzhou, 310052 People’s Republic of China

**Keywords:** Solid tumors, Immunotherapy, CAR-NK, Targeted therapy

## Abstract

Chimeric antigen receptor (CAR) represents a novel targeted therapy that uses genetic engineering to modify effector cells for precise tumor cell targeting. Chimeric antigen receptor-T (CAR-T) cell immunotherapy, which employs T cells as effectors, has demonstrated significant efficacy in treating hematologic malignancies. However, its efficacy against solid tumors remains inadequate and is accompanied by toxic side effects, including cytokine release syndrome, neurotoxicity and on-target/off-tumor effects. In contrast to T cells, natural killer (NK) cells exhibit a broader source range and can non-specifically lyse tumor cells. Moreover, it can also reduce toxicity and side effects to some extent. This review comprehensively examines recent research progress on CAR-NK therapy for solid tumors, encompassing both in vivo and in vitro studies, with a focus on CAR-NK cell design and production methods. Drawing upon laboratory and clinical evidence, this review summarizes the current challenges and side effects associated with CAR-NK technology.

**Supplementary Information:**

The online version contains supplementary material available at 10.1007/s00262-025-04106-z.

## Introduction

The CAR consists of three structural domains: an extracellular domain, a transmembrane domain and an intracellular activation domain [1]. The extracellular domain typically consists of a single-chain antibody fragment (scFv), which is primarily composed of the variable light chain (VL) and variable heavy chain (VH) of an antibody and is connected by a linker region. This scFv is further linked to the transmembrane domain via a hinge region. The scFv specifically recognizes and binds to the target antigen. The main role of the transmembrane domain is to anchor the CAR molecule to the cell membrane, thereby ensuring the stability of CAR molecule expression. The intracellular domain comprises costimulatory and signaling domains, both of which collaborate to fully activate the effector cell. Upon recognition of its specific antigen by the scFv, the transmembrane segment of the CAR transmits the ligand recognition signal to the cell interior. This activation triggers downstream pathways, ultimately leading to CAR cell activation and the subsequent killing of target cells [2].

In recent years, tumor immunotherapy technologies based on CAR-T cells have undergone rapid development and have shown excellent specificity, cytotoxicity and persistence in a multitude of clinical trials. CD19 CAR-T cell therapy has notably advanced in treating relapsed and/or refractory acute B-cell lymphoblastic leukemia (B-ALL), B-cell non-Hodgkin’s lymphoma (B-NHL) and other hematologic malignancies [3–6]. However, the therapeutic efficacy of CAR-T cells against solid tumors remains suboptimal, accompanied by toxic side effects such as cytokine release syndrome, neurotoxicity and off-target effects. To achieve efficient and safe immunotherapy for solid tumors, the exploration of novel effector cells is imperative [7].

NK cells play a crucial role in the body’s immune defense, constituting 5–15% of peripheral blood lymphocytes in humans. As innate lymphoid cells, they can directly target tumor cells in a non-specific manner. This inherent cytotoxic activity operates independently of antigen sensitization or antibody involvement and is not confined by MHC restrictions. Notably, NK cells do not secrete inflammatory factors such as interleukin (IL)-1 and IL-6 during killing, thereby avoiding cytokine storms [8]. Compared with CAR-T cells, CAR-NK cells offer advantages such as heightened tumor killing activity, reduced toxicity and a broader range of NK cell sources, positioning them as a focal point in the exploration of novel effector cells for chimeric antigen receptor therapy [9].

Despite these advantages, CAR-NK therapies have not yet received clinical approval, largely due to ongoing challenges in their development. These include limited in vivo persistence of NK cells, lower transduction and expansion efficiencies. Moreover, while preclinical data are encouraging, clinical trials demonstrating consistent efficacy and durability remain limited. These challenges have thus far limited CAR-NK cells to investigational stages, in contrast to the several CAR-T products already approved for clinical use. Addressing these hurdles is critical for the clinical translation of CAR-NK therapy, and this review aims to systematically examine the strategies being pursued to overcome them.

## CAR-NK preparation

The preparation of CAR-NK cells closely parallels the procedure employed for CAR-T cells and encompasses three pivotal stages: cell acquisition, CAR structure determination and synthesis, followed by the subsequent production and purification of CAR-NK cells.

### NK source

An essential consideration in CAR-NK therapy revolves around the procurement of NK cells, which primarily involves the isolation and expansion of NK cells sourced from peripheral blood (PB) or umbilical cord blood (UCB), the differentiation of induced pluripotent stem cells (iPSCs) into NK cells or the utilization of NK-92 cell lines [10]. In peripheral blood, NK cells constitute approximately 5–15% of lymphocytes, with CD56^dim^CD16^+^ cells representing the predominant cytotoxic subset, accounting for nearly 90% of the circulating NK population. Consequently, even with enrichment methods, the proportion of NK cells recovered from PB donors remains relatively limited. This intrinsic scarcity, combined with technical constraints in isolation and expansion, presents a significant challenge for large-scale CAR-NK cell manufacturing [11, 12]. In addition, after surgery, patients secrete more catecholamines, prostaglandins and immunosuppressive cytokines in vivo, such as transforming growth factor *β* (TGF-*β*), IL-6 and IL-10, resulting in weakened function of autologous NK cells [13–15]. More often, the source of NK cells used in adoptive therapy is often peripherally HLA-haploidentical donors [16–18]. NK-92 is the only one of the seven known NK cell lines that has consistently and repeatedly observed high antitumor cytotoxicity and can easily be genetically manipulated to recognize specific tumor antigens or to enhance monoclonal antibody activity through antibody-dependent cell-mediated cytotoxicity (ADCC). NK-92 is also the only cell line product that has been injected into patients with advanced cancer with clinical benefit and minimal side effects [19].

### Evolution of CAR structures

CAR molecules expressed on NK cells that exhibit functionality consist of three essential components: an extracellular domain, a transmembrane region and an intracellular activation moiety.

The extracellular domain comprises the antigen-binding domain and hinge region. The antigen-binding domain typically comprises a scFv sequence designed to recognize tumor-associated antigens (TAAs). The capacity of scFv to precisely target and bind to TAAs forms the cornerstone of safety and efficacy in CAR-NK immunotherapy. Ideally, TAAs should exhibit high expression levels in tumor tissue but be absent or minimally expressed in healthy tissue to mitigate off-tumor damage during treatment. Off-target damage to vital tissues or cells may pose life-threatening risks and result in treatment failure. Therefore, identifying a specific scFv for targeting TAAs is a fundamental prerequisite for successful CAR-NK therapy [20].

The hinge region comprises non-antigen-binding extracellular segments of CAR molecules. Its role is to confer flexibility, enabling the antigen-binding domain to navigate spatial obstacles and access target epitopes. Variations in the length and composition of the hinge region can impact CAR molecule expression, flexibility, signal transduction and epitope recognition, thereby influencing CAR molecule functionality. The majority of CAR-NK cells employ derivatives of the CD8*α* or CD28 extracellular structural domains or hinges derived from IgG [21].

The transmembrane domain links the extracellular domain of the CAR to its intracellular activation domain and anchors the receptor to the membrane of NK cells. In addition to this structural role, transmembrane domains can modulate CAR functionality by influencing receptor clustering and signal strength. Transmembrane segments commonly used in CAR-NK cells include those derived from CD8*α* and CD28. A comparative study by Alabanza et al. [22] demonstrated that CARs incorporating CD8*α*-derived hinge and transmembrane regions induced lower cytokine release and reduced activation-induced cell death (AICD) compared to CARs with CD28-derived segments, while maintaining comparable tumor clearance in vivo. These results suggest that the biophysical properties of the transmembrane domain may impact receptor stabilization and activation thresholds. Other transmembrane domains such as those derived from NKG2D, 2B4 and DNAM1 have also been explored in various CAR constructs [23].

The activation domain of CAR-NK cells is responsible for initiating the activation of NK cells upon recognition of target antigens. Like CAR-T cells, CAR-NK cells are categorized into four generations on the basis of the number and types of activation domains they possess [24].

First-generation CAR-NK cells, akin to CAR-T cells, incorporate only CD3*ζ* signaling. Second- and third-generation CAR-NK cells carry one and two costimulatory signals, respectively. Costimulatory molecules are typically derived from the CD28 family (such as CD28 and ICOS), the tumor necrosis factor receptor (TNFR) gene family (including 4-1BB, OX40 and CD27), or the signaling lymphocytic activation molecule (SLAM)-related receptor family (such as 2B4) [25]. To augment the functionality of CAR-NK cells, fourth-generation CAR-NK cells incorporate an intracellular domain that coexpresses specific small molecules (such as those that combine CAR expression with ectopic IL-15 expression and inducible suicide genes) [26]. This enhances NK cell proliferation further while augmenting the antitumor effects of CAR-NK cells in tumor microenvironment (TME) with limited levels of specific cytokines (such as IL-15).

While the structural components of CAR-NK cells have been well characterized, their clinical translation faces key challenges that require further investigation. The precise selection of TAAs remains critical to avoid off-tumor effects, yet antigen heterogeneity and downregulation continue to hinder consistent efficacy across different malignancies. Although the incorporation of flexible hinge regions aims to optimize antigen binding, evidence suggests that variations in hinge design may introduce trade-offs between receptor stability and functional efficiency. Similarly, while transmembrane domains such as NKG2D and CD8 improve signaling, their effectiveness depends heavily on the TME, which often requires context-specific modifications. Advances in activation domains, particularly with the inclusion of IL-15, have enhanced NK cell persistence but also present safety risks, such as overactivation and cytokine-driven toxicity. A more nuanced approach is needed—balancing flexibility, safety and efficacy through multiantigen-targeting strategies and inducible control mechanisms. Addressing these issues is essential to unlock the full therapeutic potential of CAR-NK cells and ensure their applicability across a broader spectrum of solid tumors.

### Construction, purification, expansion and cryopreservation of CAR-NK cells

Common methods for delivering the CAR sequence into NK cells to produce CAR-NK cells include viral transduction (using lentiviral or retroviral vectors), naked plasmid DNA, integration mediated by transposase DNA or mRNA electroporation transfection [27]. Lentiviruses and retroviruses are both members of the Retroviridae family. A typical feature is the ability of the RNA genome to reverse transcribe into a cDNA copy, which can then stably integrate into the host cell genome. This characteristic can lead to permanent transgene expression and relatively low inherent immunogenicity. Common lentiviral gene therapy vectors are derived from HIV-1, which has advantages such as broad infectivity, effective action on both dividing and resting cells, and long-term stable expression of exogenous genes, making it an important tool for introducing foreign genes. During host genome integration, retroviruses (such as murine leukemia viruses, MLVs) have approximately 20% of infection events occurring at the 5’ end of transcription units, with a preference for CpG islands and DNase I hypersensitive sites [28]. Indeed, early clinical trials using *γ*-retroviral vectors to treat X-linked severe combined immunodeficiency (SCID-X1) resulted in T cell leukemia in several patients due to vector insertion near oncogenes such as LMO2, BMI1 and CCND2 [29]. These findings underscore the potential carcinogenic risk associated with retroviral vectors. In contrast, lentiviral vectors mainly integrate into sites far from the transcription start site. Therefore, compared with retroviral vectors, lentiviral vectors have a lower potential for carcinogenesis and are safer for clinical use, making them the most commonly used viral transduction method.

Following transduction, CAR expression must be promptly validated to ensure quality and consistency of the therapeutic product. Surface CAR expression is typically assessed by flow cytometry using fluorophore-conjugated anti-tag antibodies (e.g., anti-myc, anti-Fab) or antigen-based probes targeting the scFv. In particular, Protein L—which binds to the kappa light chain variable region without interfering with antigen binding—has emerged as a robust, tag-independent detection reagent, suitable for a wide range of CAR constructs [30] [31]. Alternatively, soluble antigen probes such as CD19-Fc [31] or GPC3-Fc [32] can detect functional CARs with intact binding capacity. These detection strategies are essential not only for quality control but also for downstream purification. Since transduced NK cell populations typically contain both CAR-expressing and unmodified cells, enrichment steps—such as magnetic bead isolation or fluorescence-activated cell sorting (FACS)—are required to isolate the CAR-positive fraction. This purification step is critical to ensure a uniform and potent therapeutic product, prevent dilution of CAR-specific cytotoxicity by untransduced cells and enable accurate dosing and batch standardization for clinical application.

In addition to stable integration methods such as lentiviral or retroviral transduction, electroporation of CAR-encoding mRNAs represents a rapid and non-integrating approach for engineering CAR-NK cells. This method avoids risks associated with genomic integration and is particularly suitable for applications requiring transient expression. However, due to the short-lived nature of mRNA, CAR expression in electroporated NK cells typically lasts only a few days. As such, CAR-NK cells generated by mRNA electroporation must be infused fresh—without cryopreservation—within approximately 7 days of production. Cryopreservation is generally avoided during this window, as freeze–thaw processes may further diminish CAR surface expression and compromise functional activity. This approach is commonly used in clinical studies aiming to balance rapid production timelines with acceptable safety profiles for short-term therapies [2].

The expansion rate of CAR-NK cells is a crucial aspect of CAR-NK cell therapy, as it directly impacts the feasibility of obtaining sufficient NK cells from limited donor material. To enhance proliferation and functionality, feeder cell systems are commonly employed, including Epstein–Barr virus-transformed lymphoblastoid cell lines (EBV-LCL) and the K562 myelogenous leukemia cell line [33]. Among these, a B-cell line transformed with EBV, 721.221, has been engineered to express membrane-bound IL-21 (221-mIL-21) [34] [35]. IL-21 is particularly effective in driving NK cell proliferation, making this system ideal for achieving high expansion fold and purity. Studies have shown that the 221-mIL-21 feeder system supports robust NK expansion from diverse sources, with NK cell purity reaching 90% and T cell contamination remaining below 5% after 14 days of post-expansion culture.

Alternatively, the K562 cell line has been modified to express membrane-bound IL-2 (mbIL2) or IL-13 (mbIL13) [36]. Unlike IL-21, which primarily promotes proliferation, IL-2 and IL-13 provide distinct activation signals. NK cells cultured with K562-mbIL2 or mbIL13 feeders exhibit enhanced expression of activating receptors such as NKG2D and show increased cytotoxic activity against tumor cells compared to NK cells cultured with unmodified K562 cells. These findings suggest that IL-2 or IL-13 stimulation is particularly advantageous when the goal is to enhance NK cell activation and effector function rather than sheer proliferation.

Therefore, the choice of feeder cell system depends on the desired outcome: IL-21-based feeders are optimal for maximal expansion with high purity, while IL-2 or IL-13-based feeders are preferred when boosting cytotoxic function is prioritized. In some cases, combining multiple cytokine signals may be explored to simultaneously enhance both expansion and activation phenotypes.

**Fig. 1:**
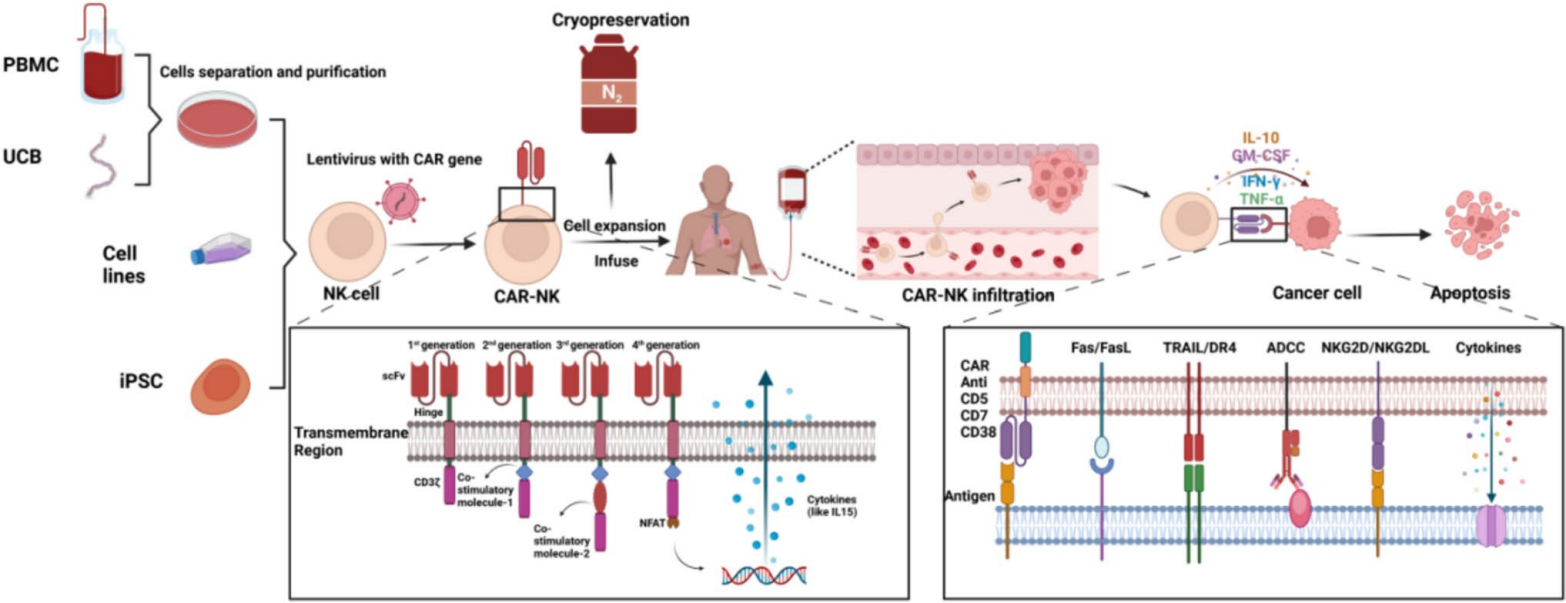
Preparation of CAR-NK cells and their recognition and killing of tumor cells. Different sources of NK cells are infected with lentivirus to generate different generations of CAR-NK cells, which are then infused back into the human body to target tumors and kill tumor cells through various means, including CAR-mediated targeting, Fas/FasL interactions, TRAIL/DR4 pathways, ADCC, and NKG2D/NKG2DL interactions. This figure was drawn using Biorender

Additionally, cryopreservation plays a crucial role in providing readily available CAR-NK cell products, thus garnering significant attention. Dimethyl sulfoxide (DMSO) is commonly used in cryopreservation processes. However, its utilization has been associated with notable clinical side effects, including cardiovascular, neurological, gastrointestinal and allergic reactions [37], in patients receiving cell therapy product infusions. To solve this problem, many teams have modified the composition of the cryopreservation solution and invented a variety of new DMSO-free cryopreservation media, and it has been proven that NK-92 cells still maintain cytotoxic activity after long-term cryopreservation in new DMSO-free media [38–40]. Recent research has also focused on intrinsic cellular damage mechanisms associated with cryopreservation. A study by Berjis et al. (2024) [41] revealed that cryopreserved NK cells undergo granzyme B (GZMB)-mediated autolysis due to intracellular leakage from cytotoxic granules. Pretreatment with IL-15 and IL-18 prior to freezing was shown to induce transient degranulation and enhance expression of anti-apoptotic genes such as BCL2L1, effectively reducing post-thaw apoptosis and preserving NK cell viability and function. A schematic representation of CAR-NK cell preparation, tumor recognition and killing by CAR-NK cells is shown in Fig. 1.

While various CAR sequence delivery methods offer unique benefits, each presents challenges that impact clinical application. Lentiviral transduction provides stable gene expression with low immunogenicity, making it a preferred approach, although safety concerns around insertional mutagenesis persist. mRNA electroporation offers a rapid, non-integrative solution but is limited by the transient nature of expression, necessitating immediate administration. Feeder cell-based expansion using lines such as K562-mbIL2 and 221-mIL-21 significantly enhances NK cell proliferation and functionality, although variability in expansion rates limits reproducibility across samples. Cryopreservation, which is essential for off-the-shelf CAR-NK products, faces toxicity challenges associated with DMSO, driving the development of safer, DMSO-free media. However, maintaining long-term NK cell functionality without compromising safety remains a work in progress. Future efforts must focus on refining delivery and expansion techniques, optimizing cryopreservation strategies and ensuring consistency in large-scale production to fully unlock the therapeutic potential of CAR-NK cells in clinical settings.

## Research progress on CAR-NK therapy for solid tumors

In recent years, CAR-NK cell therapy has been extensively tested in tumors such as pancreatic cancer, breast cancer and glioblastoma, confirming its therapeutic efficacy in treating solid tumors. A schematic representation of the targets of CAR-NK cells in different solid tumors is presented in Supplementary Fig. 1.

### Pancreatic cancer (PC)

PC ranks as the third leading cause of cancer-related fatalities in females (8%) and the fourth leading cause in males (8%) [42]. It frequently eludes early detection because of its non-specific symptoms and deep anatomical location, compounded by the absence of effective early screening methods [43]. Even with treatments such as surgery, radiotherapy and chemotherapy, the prognosis remains grim, with a mere 10% 5-year survival rate. The distinctive TME of PC, characterized by dense connective tissue growth and high levels of immunosuppressive cell infiltration, poses formidable challenges for the treatment of advanced PC [44].

In pancreatic ductal adenocarcinoma (PDAC), folate receptor alpha (FR*α*) and death receptor 4 (DR4) are significantly overexpressed and are typically associated with a poor prognosis [45]. Therefore, they also represent potential targets for biological therapy. Researchers reprogrammed allogeneic FR*α*-targeted CAR-NK cells to express ligands that induce apoptosis. These modified CAR-NK cells initiate DR4/5-mediated selective cell death in tumors positive for FR*α* and DR4/5, leading to considerable apoptosis of tumor cells [46].

The expression of prostate stem cell antigen (PSCA) is specifically elevated in primary human pancreatic cancer cells compared with adjacent normal tissue [47]. Using primary human NK cells derived from umbilical cord blood, a CAR-NK-targeting PSCA was engineered. These cells, along with ordinary NK cells, were separately cocultured with the PSCA antigen-positive human pancreatic cancer cell line Capan-1 and the PSCA antigen-negative human pancreatic cancer cell line PANC-1 in vitro. The secretion levels of IFN-*γ* during NK cell activation were assessed via ELISA. The results revealed a significant increase in IFN-*γ* secretion in the coculture group of PSCA-CAR-NK cells and Capan-1 cells, accompanied by a notable decrease in the survival rate of Capan-1 cells. Additionally, PSCA-CAR-NK cells demonstrated remarkable therapeutic efficacy in a pancreatic cancer mouse model transplanted with Capan-1 cells, leading to significant suppression of tumor growth without severe toxic side effects. These findings provide a solid theoretical basis for further clinical applications [44].

Mesothelin (MSLN) is a protein highly expressed on the surface of various solid tumors, including pancreatic cancer cells, but rarely or not at all on normal cells. This target has been repeatedly used to treat pancreatic cancer with CAR-T cells, and its effectiveness has been verified [48–51]. Researchers constructed MSLN-targeted CAR-NK cells and reported that MSLN-CAR-NK cells, in combination with a stimulator of interferon genes (STING) agonist, could effectively eliminate MSLN-positive human pancreatic cancer cells in the AsPC-1 cell line. In vivo studies further demonstrated that MSLN-CAR-NK cells significantly suppressed tumor growth in the AsPC-1 mouse transplant model, resulting in prolonged survival. These findings suggest that the combined application of STING agonists and CAR-NK cells could offer a promising immunotherapy approach for pancreatic cancer [52].

CD70 is expressed at low levels in healthy tissues but plays a significant role in the progression of many malignant tumors [53–55], making it a potential therapeutic target for treating PDAC. CAR-NK cells targeting CD70 were engineered and endowed with the ability to secrete IL-15. The combined action of IL-15 and CAR-NK effectively eliminated CD70-positive tumor cells and increased the survival rate of mice harboring CD70-positive tumors. The team suggested that IL-15 stimulation enhances the efficacy of CD70-CAR-NK cells by increasing CAR expression and increasing the secretion of proinflammatory cytokines (mainly through autocrine or intracellular pathways) [56].

While CAR-NK-cell therapies targeting pancreatic cancer cells have promising potential, significant challenges remain. The heterogeneity of the TME and the variable expression of antigens such as PSCA, MSLN and CD70 across patient populations underscore the need for personalized approaches. The synergistic effect of STING agonists with MSLN-CAR-NK cells represents an innovative strategy to increase antitumor immunity, although the precise modulation of immune responses to prevent potential adverse effects needs exploration. Similarly, CD70-targeted CAR-NK cells armed with IL-15 offer enhanced cytotoxicity, but their application is tempered by the need to manage cytokine-related toxicity and ensure sustained efficacy in immunosuppressive microenvironments. Future research should prioritize optimizing CAR constructs, strategically integrating immunotherapies and refining patient stratification to translate promising findings into effective treatments, particularly for pancreatic cancer patients with advanced or resistant disease.

### Breast cancer (BC)

BC arises from the uncontrolled proliferation of mammary epithelial cells influenced by various carcinogenic factors [57]. BC is estimated to constitute the most common new cancer in women in 2024, at approximately 32% [42]. CAR-NK cells targeting human epidermal growth factor receptor 2 (HER2) recognize all breast cancer cells expressing HER2 and exhibit potent antitumor activity both in vitro and in vivo [58]. Furthermore, antiestrogen therapy is the primary treatment for estrogen receptor (ER)-positive breast cancer, with ER inhibitors serving as first-line antiestrogens for the refractory phenotype of advanced cancer, acting by modulating ER transcriptional function [59, 60]. Fulvestrant currently stands as the sole clinically approved pure antiestrogen capable of inducing ER degradation. However, tumors frequently develop resistance to fulvestrant [61–63], leaving chemotherapy as a late-line treatment option for fulvestrant-resistant tumors, with few alternatives available. A study showed that fulvestrant resistance coincides with increased expression of numerous innate immune response genes, including the cell surface NK cell NCR3 ligand B7-H6. NCR3 (NKp30), a membrane receptor on NK cells, binds to its ligand B7-H6, thereby triggering NK cell activation. This activation is mediated by coupling NKp30, which has no major intracellular domain for activation signaling, with CD3*ξ* and Fc*ε*RI*γ* in trans through opposing charge contacts within the corresponding transmembrane domains of these proteins [64]. To address this, researchers devised a chimeric molecule in which NKp30 comprises its extracellular ligand-binding domain, transmembrane domain and intracellular domain linked to CD3. CD3 harbors four immunoreceptor tyrosine-based activation motifs (ITAMs) for CAR-NK cell immune activation. These findings demonstrated that CAR-NK cells targeting B7-H6 exhibit greater specific killing of fulvestrant-resistant breast cancer cells than regular NK cells do, underscoring the promise of CAR-based cell therapy in overcoming endocrine-resistant breast cancer [65].

Triple-negative breast cancer (TNBC), distinguished by the absence of ER, progesterone receptor (PR) and HER2 expression, comprises approximately 15% of all diagnosed breast cancers worldwide. The absence of identifiable surface markers presents a considerable challenge in treating TNBC, making it challenging to manage and increasing the risk of recurrence [66].

Tissue factor (TF) is a surface target present in 50–85% of TNBC patients and holds significant promise as a therapeutic target. CAR-NK cells designed to target TF have demonstrated encouraging antitumor effects in both TNBC cell lines and TNBC xenograft mouse models [67].

Recently, a group pioneered the development of CD44v6-targeted CAR-NK cells, which exhibited potent cytotoxicity against CD44v6-positive TNBC cell lines, such as MDA-MB-231, MDA-MB-468 and HCC1937, in vitro. Furthermore, these CD44v6-targeted CAR-NK cells were assessed in a 3D TNBC multicellular tumor spheroid (MCTS) model, where they exhibited effective cytotoxicity against TNBC within 3D spheroids. This breakthrough presents novel treatment prospects for breast cancer, including TNBC [66].

Another team developed a TNBC xenograft model in zebrafish larvae and generated two CAR-NK cell lines targeting PD-L1 and ErbB2 to combat MDA-MB-231 breast cancer cells expressing PD-L1 and MDA-MB-453 breast cancer cells expressing ErbB2. In the zebrafish xenograft model, injected cancer cells populated the peripheral areas of the larvae, including the caudal hematopoietic tissue (CHT), mirroring cancer cell homing to hematopoietic sites. CAR-NK cells injected 2.5 h later displayed mild in vivo cytotoxicity compared with unmodified NK-92 cells. These CAR-NK cells migrated to CHT and promptly eliminated individual cancer cells throughout the organism. Additionally, confocal live-cell imaging revealed vascular intravasation and real-time interaction of CAR-NK cells with MDA-MB-231 cells, elucidating the rapid and effective in vivo cytotoxicity of CAR-NK cells. This finding also illustrated that zebrafish larvae can serve as a rapid and cost-effective in vivo platform for evaluating CAR-NK cell efficacy and predicting patient response to treatment [68].

Epidermal growth factor receptor (EGFR), also known as HER1, is overexpressed in at least 20% of breast cancers and is associated with poor prognosis in patients [69]. HER1-targeted CAR-NK therapy has been a promising approach for the immunotherapy of TNBC. In a pioneering study, the H_2_O_2_ enzyme-encoding gene was introduced into HER1-CAR-NK cells for the treatment of TNBC. This study demonstrated that HER1-CAR-NK cells could continuously alleviate tumor hypoxia and exhibited significantly enhanced persistence and effector function within TNBC tumors. Local therapy with HER1-CAR-CAT-NK cells not only inhibited the growth of primary residual tumors but also triggered systemic antitumor responses, suppressing the growth of distant tumors [70].

Future research on CAR-NK therapy for breast cancer, especially TNBC, must address tumor heterogeneity and overcome immunosuppressive microenvironments. While targeting antigens such as TF and CD44v6 has shown promise, variability in antigen expression highlights the need for refined patient stratification and optimized CAR designs to increase selectivity and reduce off-target effects. CD44v6-specific CAR-NK cells armed with IL-15 superagonists and checkpoint inhibitors have demonstrated resistance to immunosuppression, whereas TF-targeted CAR-NK cells have exhibited robust cytotoxicity and ADCC potential. Additionally, B7-H6-targeting CAR-NK cells provide an innovative solution for fulvestrant-resistant breast cancers by leveraging NK cell activation. Future efforts should focus on integrating CAR-NK therapy with complementary immunotherapies, improving preclinical models for validation and developing scalable production strategies to ensure clinical applicability and long-term therapeutic success.

### Glioblastoma (GBM)

GBM is the most malignant form of astrocytoma and is characterized by low tumor cell immunogenicity, invasive growth that poses challenges for complete surgical removal and a generally immunosuppressive TME [71]. It is an aggressive primary tumor that causes thousands of deaths worldwide every year. The mean survival of patients with GBM remains less than 20 months despite currently available therapies [72]. Nevertheless, approximately 89% of GBMs exhibit natural infiltration by NK cells, suggesting the feasibility of transporting CAR-NK cells into tumor tissues [73].

EGFRvIII, a common oncogenic variant of EGFR present in 30 ~ 40% of malignant gliomas, significantly contributes to tumorigenicity owing to its structurally activated kinase domain [74, 75]. EGFRvIII-targeted CAR-NK cells that overexpress CXCR4 receptors were developed to enhance binding to gliomas during adoptive transfer. These EGFRvIII-targeted CAR-NK cells effectively eradicated EGFRvIII-positive human glioblastoma cell lines, namely, U87-MGEGFRvIII and BS153resE. In in vivo experiments, marked suppression of GBM tumor growth and prolonged survival were observed in a mouse model of GBM [76].

As early as 2016, a study revealed the potent and selective antitumor activity of CAR-NK cells, which target the growth factor receptor tyrosine kinase ErbB2 (HER2), against GBM cells in both in vitro and in situ GBM xenograft models [77].

Recently, researchers administered HER2-targeted CAR-NK cell therapy to 9 recurrent HER2-positive GBM patients during recurrent surgery, injecting them into the surgical cavity margins. Follow-up imaging, peripheral blood lymphocyte phenotypic analysis and analysis of immune structures through multiplex immunohistochemistry and spatial digital analysis were conducted. Therapy showed no dose-dependent toxicity, and none of the patients experienced cytokine release syndrome or immune effector cell-related neurotoxicity syndrome. Five patients remained stable after recurrent surgery and CAR-NK injection for 7–37 weeks, whereas four patients experienced disease progression. Pseudoprogression at the injection site was observed in 2 patients, indicating a treatment-induced immune response. The median progression-free survival for all patients was 7 weeks, and the median overall survival was 31 weeks. Additionally, the level of CD8^+^ T cell infiltration in recurrent tumor tissue before CAR-NK-cell injection was positively correlated with the time to progression. The team concluded that intracranial injection of HER2-targeted CAR-NK cells is feasible and safe in recurrent GBM patients. The maximum feasible dose for subsequent expansion cohorts was determined to be 1 × 10^8^ CAR-NK cells, and repeated local injections of CAR-NK cells should be considered [78]. The disialoganglioside GD2 (GD2) is highly expressed in neuroblastoma and most melanomas and is variably expressed in a range of other tumors, including bone and soft tissue sarcomas, small cell lung cancer and brain tumors, making it a promising target for immunotherapy research [79]. However, clinical trials targeting GD2 with CAR-T cells have shown limited efficacy [80, 81]. Other researchers also developed two patient-derived xenograft models of diffuse intrinsic pontine glioma (DIPG) to assess the antitumor efficacy of GD2-CAR-NK-92 cells in vivo. The cytotoxicity of GD2-CAR-T cells to NK-92 cells was first evaluated in vitro. In vivo experiments demonstrated that GD2-CAR-NK-92 cells inhibited tumor growth and prolonged overall survival in a group of patient-derived xenografted mice with high GD2 expression [82].

Future investigations should prioritize optimizing CAR-NK-containing constructs and integrating immunotherapies that target the immunosuppressive microenvironment of glioblastoma. Strategies such as enhancing homing with chemokine receptors such as CXCR4 or employing CAR-NK cells expressing IL-15 to increase persistence have shown promising preclinical efficacy. Additionally, novel antigen-specific approaches, such as EGFRvIII-targeted CARs, offer hope but remain limited by antigen heterogeneity and tumor escape mechanisms. Early clinical trials demonstrated the feasibility and safety of HER2-targeted CAR-NK cells, although therapeutic durability remains an obstacle, as evidenced by mixed outcomes in recurrent GBM patients. Overcoming these challenges will likely require combinatorial approaches, including repeated local administrations and synergistic use of immune checkpoint inhibitors. These advancements are crucial for translating CAR-NK cell therapy into a viable option for GBM treatment, especially given the dire prognosis associated with this aggressive malignancy.

### Ovarian cancer (OC)

Ovarian cancer, one of the most prevalent and lethal gynecological malignancies, often remains undetected until advanced stages, leading to a lack of effective early diagnosis. OC is estimated to be the sixth leading cause of cancer-related death among women in 2024, or approximately 4% [42]. Consequently, there is an urgent need for innovative therapeutic strategies to improve patient outcomes [83].

MSLN is overexpressed in ovarian cancer, making it an ideal target for immunotherapy [84]. Zhang’s team devised MSLN-targeted CAR-NK cells, which demonstrated robust and specific cytotoxicity against MSLN-positive human ovarian cancer cell lines, namely, OVCAR-3 and SKOV3. These cells effectively prevent the growth of subcutaneous and intraperitoneal ovarian cancer in animal models [85].

Furthermore, engineered CD24-targeted CAR-NK cells effectively and specifically killed the CD24-positive ovarian cancer cell lines OVCAR-3 and SKOV3 both in vitro and in vivo [86]. FR*α*-targeted CAR-NK cells also exhibited high cytotoxicity against ovarian cancer cells. Three generations of anti-FR*α*-CAR-T cells, namely, FR*α*–*ζ* (first generation), FR*α*–28*ζ* (second generation) and FR*α*–28BB*ξ* (third generation), all of which selectively eliminate the FRα-positive human ovarian cancer cell lines SK-OV-3 and A2780 [87].

CAR-NK therapies targeting ovarian cancer, with MSLN-, CD24- and *α*FR-targeted CAR-NK cells demonstrating strong preclinical efficacy, show promise. However, challenges remain in ensuring persistence and overcoming immunosuppressive microenvironments.

### Colorectal cancer (CRC)

CRC ranks as one of the most prevalent malignant tumors and is estimated to be the third most common cancer in 2024 (8%) [42], and its treatment options include surgery, chemotherapy, radiotherapy and immunotherapy. While surgery serves as the primary treatment, chemotherapy, which is vital for restraining tumor growth and metastasis, frequently falls short in curing patients with advanced disease or post-surgical recurrence [88].

Epithelial cell adhesion molecule (EpCAM, CD326) is overexpressed in many solid tumors but is expressed at low levels in normal epithelium. Elevated EpCAM expression is detected in 97.7% of colorectal adenocarcinoma patients [89]. Researchers engineered EpCAM-targeted CAR-NK cells, which exhibited specific cytotoxicity against EpCAM-positive human colorectal cancer cell lines, namely, HCT-8 and HCT-116. Additionally, they demonstrated effective antitumor activity in a NOD/SCID mouse model of colorectal cancer in which HCT-8-Luc cells were transplanted [90].

In 2018, CAR-NK-92, which significantly increased the cytotoxicity against CEA-positive colon cancer cell lines, was developed to target carcinoembryonic antigen (CEA) [91]. In 2024, a novel generation of CAR-NK cells targeting CEA was developed. This advancement involves combining PD-1 checkpoint inhibitors and CCR4 chemokine receptors to augment the homing and infiltration of CAR-NK cells within the TME. By utilizing a sophisticated 3D multicellular tumor spheroid model (MCTS) that replicates key TME elements, CEA-CAR-NK-92 cells exhibited potent cytotoxicity against CRC cell lines and MCTS models. The precision of this therapy is underscored by its low specificity against non-cancerous cell lines. Furthermore, the incorporation of CCR4 migration receptors facilitates CAR-NK cell homing by recognizing the target ligands CCL17 and CCL22 [92].

In addition, as mentioned above, CAR-NK cells target CD70, which targets tumor cells in CRC patients and induces significant specific killing [56].

CAR-NK therapies for CRC show significant potential, with EpCAM- and CEA-targeted CAR-NK cells demonstrating effective tumor suppression. The incorporation of chemokine receptors such as CCR4 enhances cell homing and infiltration into the TME, increasing therapeutic outcomes. Synergistic combinations with checkpoint inhibitors, such as PD-1 blockers, and chemotherapy agents, such as regorafenib, offer promising strategies to overcome immune evasion and resistance. Further optimization of CAR design, improved persistence within the TME and mitigation of off-target effects are essential to achieve durable clinical responses.

### Lung cancer

Lung cancer, a leading cause of cancer-related death, could be the most dangerous cause of cancer-related death in men in 2024 [42] and encompasses various histological subtypes, including small cell lung cancer (SCLC), lung adenocarcinoma (LUAD) and non-small cell lung cancer (NSCLC). Owing to late-stage diagnosis in 75% of patients, the 3-year survival rate remains below 20%. Research focused on specific upregulated proteins in different lung cancer cells has revealed numerous potential therapeutic targets [93].

SCLC, renowned for its high recurrence and drug resistance, frequently overexpresses Delta-like ligand 3 (DLL3). DLL3-targeted CAR-NK92 cells display effective antitumor activity against DLL3-positive SCLC cells [94]. In LUAD, the overexpression of mesenchymal epithelial transition factor (C-Met) represents a recognized therapeutic target. c-Met-CAR-NK cells effectively target the C-Met-positive lung cancer cell line H1299 [95]. In a recent study, CAR-NK cells coexpressing NKG2D and IL-21 were established, and their efficacy in treating lung cancer was evaluated both in vitro and in vivo. Researchers have reported that IL-21 expression significantly enhances the cytotoxicity of NKG2D CAR-NK cells against lung cancer cells in a dose-dependent manner. Moreover, IL-21 inhibited tumor growth both in vitro and in vivo. Additionally, the proliferation of NKG2D-IL-21 CAR-NK cells was augmented, whereas the apoptosis and exhaustion of these CAR-NK cells were suppressed. The team proposed that IL-21-mediated NKG2D CAR-NK cell functionality operates via the activation of the AKT signaling pathway [96].

Although these approaches show encouraging results, further work is needed to optimize the tumor infiltration of CAR-NK cells, sustain therapeutic effects and ensure broader clinical applicability.

### Hepatocellular carcinoma (HCC)

HCC may be the fifth leading cause of cancer-related death in men in 2024 [42]. HCC constitutes more than 90% of liver cancers and progresses through a multifaceted process. Upon development, HCC cells demonstrate robust invasiveness, and existing treatment options are scarce, resulting in a dismal five-year survival rate of less than 20%. The quest for suitable and efficacious treatment modalities is urgently needed [97, 98].

Glypican-3 (GPC3), a member of the heparan sulfate proteoglycan family, is one of the most extensively studied TAAs in HCC. Its restricted expression in normal adult tissues and overexpression in most HCCs make it a logical target for CAR-based immunotherapy against HCC [61, 99]. GPC3-targeted CAR-NK cells have demonstrated strong antitumor activity in vitro and in animal models, effectively infiltrating tumor sites, reducing proliferation` and inducing apoptosis. Efforts to overcome the immunosuppressive liver microenvironment, such as co-targeting the TGF-*β* signaling pathway, have further enhanced the therapeutic potential of GPC3-directed CAR-NK therapies. Efforts to overcome the immunosuppressive liver microenvironment, such as co-targeting the TGF-*β* signaling pathway, have further enhanced the therapeutic potential of GPC3-directed CAR-NK therapies [100].

In addition to GPC3, CD147 (also known as basigin or EMMPRIN) has recently emerged as another promising immunotherapeutic target in HCC. Liu and colleagues developed a CAR construct targeting CD147 and demonstrated that both CD147-CAR-T and CD147-CAR-NK cells effectively eliminated HCC cell lines and xenografts in vitro and in vivo [101] To improve antigen specificity and minimize off-tumor toxicity, the authors further designed a logic-gated GPC3–synNotch–inducible CD147-CAR system, which selectively killed dual-positive (GPC3⁺CD147⁺) tumor cells while sparing CD147⁺ normal cells.

Subsequently, the same team established a human CD147 knock-in NSG mouse model to investigate both SARS-CoV-2 infection biology and CD147-targeted immunotherapies. While originally designed to explore viral entry mechanisms, this transgenic model provided a physiologically relevant platform for evaluating immune-based interventions involving CD147 [102].

More recently, Sabha et al. compared CD147-CAR-T and CD147-CAR-NK cells in the human CD147 knock-in HCC model and found that CAR-NK cells maintained high antitumor efficacy with minimal toxicity to CD147⁺ healthy tissues and reduced neuroinflammation compared to CAR-T cells. This study supports the potential safety advantages of CD147-targeted CAR-NK therapy in clinical settings [103].

Together, these findings highlight the importance of both GPC3 and CD147 as clinically relevant antigens in the context of HCC and justify the continued exploration of dual-antigen targeting and logic-gated CAR-NK strategies.

### Prostate cancer (PCa)

PCa ranks as the most common cancer in men, accounting for 29% of new cancer cases in 2024 [42]. Androgen deprivation therapy (ADT) is widely utilized because of its association with abnormal androgen and androgen receptor (AR) signaling in the majority of prostate cancers. Nevertheless, most patients eventually develop resistance to ADT, progressing to castration-resistant PCa (CRPC) or metastatic CRPC (mCRPC), with a median survival of approximately 14 months [104].

Prostate-specific membrane antigen (PSMA) levels can distinguish between normal and cancerous prostate tissues and are correlated with Gleason scores. Researchers engineered PSMA-targeted CAR-NK cells, which demonstrated specificity and cytotoxicity against the PSMA-positive prostate cancer cell line LNCaP, effectively impeding tumor growth and enhancing survival in mouse models [105].

PSMA-targeted CAR-NK cells offer a promising strategy for PCa, particularly for addressing CRPC and mCRPC. Unlike ADT, which is limited by AR-driven resistance, CAR-NK therapies provide an innovative, off-the-shelf approach with lower toxicity and strong antitumor potential. Enhancing CAR-NK persistence and integrating them with therapies targeting AR signaling or immune checkpoints will be essential for overcoming resistance and achieving durable clinical responses in advanced PCa patients.

### Squamous cell carcinoma (SCC)

Squamous cell carcinoma is a malignant tumor that arises in the skin, adnexa or mucosa [106, 107]. Currently, CAR-NK therapy has been employed in the treatment of esophageal squamous cell carcinoma (ESCC) and oral tongue squamous cell carcinoma (OTSCC).

CD276 exhibited positive staining in 51.43% (54/105) and 51.35% (38/74) of stage III and IV esophageal squamous cell carcinoma (ESCC) patients, respectively, across all disease stages. Using pluripotent stem cell-derived NK cells, the team engineered CD276-targeted CAR-expressing NK cells and assessed their cytotoxicity against ESCC via a patient-specific organoid (PSO) model. These CD276-targeted CAR-NK cells demonstrated specific and significant cytotoxic activity against CD276-positive ESCC PSOs and exhibited substantial cytotoxicity in a murine model [108]. Cripto-1 [109] and CD22 [110] have also emerged as potential targets for CAR-NK cell therapy in ESCC.

Furthermore, the same group reported MUC1 expression in 79.5% (66/83) and 72.7% (24/33) of stage III and IV OTSCC patients, respectively. In response, the group generated induced pluripotent stem cell-derived MUC1-targeting CAR-NK cells and evaluated their efficacy against human OTSCC in vitro and in a mouse xenograft model [111].

CAR-NK-cell therapies targeting SCC constitute a novel approach by addressing the limitations of conventional treatments, but several challenges must be overcome to ensure clinical success. The use of pluripotent stem cell-derived CAR-NK cells, as seen with CD276 and MUC1 targets, provides a scalable and consistent therapeutic option. However, the heterogeneity of SCCs, particularly ESCC and OTSCC, raises concerns about antigen escape, underscoring the need for multitarget strategies, such as combining CD22 or Cripto-1 with primary targets. Furthermore, while preclinical models highlight the cytotoxic potential of these therapies, the immunosuppressive TME remains a significant barrier to sustained efficacy. Increasing CAR-NK cell persistence, possibly through genetic modifications or cytokine support systems, will be critical to maintaining therapeutic pressure over time. Integrating these therapies with immune checkpoint inhibitors or other systemic treatments could also increase their effectiveness. These strategies will be essential for translating the preclinical success of CAR-NK therapies into durable, real-world outcomes for SCC patients.

### Challenges of CAR-NK cell therapy in solid tumors

Despite the encouraging progress of CAR-NK cell therapy across various solid tumors, including pancreatic, breast, glioblastoma, ovarian, colorectal, lung and prostate cancers, several shared challenges continue to limit its clinical translation. Many of these issues mirror those observed in CAR-T cell therapy and remain only partially resolved in CAR-NK applications. One major hurdle is the inefficient trafficking and infiltration of CAR-NK cells into solid tumor sites, often impeded by the dense extracellular matrix, abnormal tumor vasculature and chemokine mismatches between the tumor and infused NK cells. In addition, the immunosuppressive TME—rich in inhibitory cytokines such as TGF-*β* and IL-10, regulatory cells, hypoxia and metabolic suppressors like adenosine—can significantly impair CAR-NK cell survival and effector function. Tumor antigen heterogeneity and loss also pose major obstacles, leading to immune escape when therapies rely on single-target CAR constructs. Furthermore, CAR-NK cells typically display limited in vivo persistence, especially in the absence of cytokine support or engineered survival signals, thereby reducing the durability of antitumor responses. Addressing these shared barriers is essential for advancing CAR-NK therapies from promising preclinical models to durable, effective treatments in the clinic. A more detailed discussion of current and emerging strategies to overcome these challenges is provided in Sect. "Evaluation of the application prospects of CAR-NK cells.”

## Clinical potential and advantages of CAR-NK therapy in solid tumors

The efficacy of CAR-T cell therapy in treating hematological malignancies has been well established. However, unlike in hematological cancers, the success of CAR-T cell therapy in solid tumors is limited by the ability of these cells to transport and infiltrate these tumors. Physical barriers, such as cancer-associated fibroblasts (CAFs) and abnormal vasculature at the tumor site, can obstruct T cell entry [112]. The presence of high endothelial venules (HEVs) is crucial for T cell infiltration, but these vessels are often distorted or immature in many solid tumors, further complicating CAR-T cell infiltration [113]. Moreover, adoptively transferred CAR-T cells can proliferate and persist in the body, leading to the release of large amounts of inflammatory cytokines such as TNF-*α*, IL-6 and IL-1 and finally cytokine release syndrome (CRS), which can result in complications such as capillary leak syndrome (CLS), disseminated intravascular coagulation (DIC) and even immune effector cell-associated neurotoxicity syndrome (ICANS), thereby limiting the clinical application of CAR-T cell therapy [114]. CRS is widely regarded as the most severe complication of CAR-T cell therapy and poses a significant threat to life [97, 98]. The clinical manifestations of CRS include organ dysfunction, such as hyperthermia, sinus tachycardia, hypotension, hypoxia and cardiac insufficiency [115–119]. Current evidence suggests that when CAR-T cells exert cytotoxic effects on tumor cells, the overproduction of proinflammatory cytokines, primarily IL-1, IL-6 and TNF-*α*, drives the development of CRS. These cytokines, in turn, activate NK cells, endothelial cells, monocytes/macrophages and dendritic cells, further amplifying the secretion of IL-6, IL-1, IL-5, IL-10 and other cytokines in a cascading reaction. This leads to a severe systemic inflammatory response [116]. The mechanism by which CAR-T cell therapy causes CRS is shown in Supplementary Fig. 2.

ICANS, while slightly less severe than CRS, generally has a better prognosis and is often reversible [120]. Clear evidence now indicates that ICANS is closely related to systemic cytokine levels and CRS severity. Cytokines such as IFN-*γ*, IL-15, IL-6, IL-10, GM-CSF, IL-2, IL-2R*α*, IL-1RA and CXCL10 are associated with ICANS. However, owing to the lack of a definitive pathophysiological connection, not all patients with severe CRS develop ICANS, nor do all patients with ICANS experience CRS [120]. Therefore, ICANS is described as a condition “associated with the onset of CRS but independent of it” [6]. In light of these concerns, safer and more effective CAR-NK therapies are gaining increased attention. CAR-NK cells are associated with fewer and less severe cases of CRS, likely due to their distinct activation pathways and antitumor mechanisms (dominated by IFN-*γ* and GM-CSF). Furthermore, CAR-NK cell infusion reduces the risk of GvHD [10, 27, 121–123]. Below is a compilation of clinical trials involving CAR-NK cells for antitumor therapy, as registered on ClinicalTrials.gov (Table 1). In the trial NCT03415100, CAR-NK cells demonstrated a potent and specific cytotoxic effect against NKG2D ligand-positive HCT116 human colorectal cancer cells. None of the three patients experienced severe adverse events (≥ grade 3). Only grade 1 CRS was observed, presenting with fever, fatigue and reduced appetite. Fever was associated with elevated plasma levels of interleukin-6 (IL-6) (224 pg/mL) and C-reactive protein (CRP) (117.6 mg/L). The symptoms subsided after the application of an ice pack. Additionally, no cases of GvHD were observed in two patients treated with haploidentical NK cells [124].

**Table 1:** Clinical studies of CAR-NK treatment in solid tumors (Data source: clinicaltrials.gov)

Disease	Targeted	Sponsor	First posted	Clinicaltrial no
Ovarian epithelial carcinoma	MESO	Allife Medical Science and Technology Co., Ltd	2018.1	NCT03692637
	MESO	Shantou University Medical College	2023.6	NCT05856643
Ovarian cancer	NKG2D	Hangzhou Cheetah Cell Therapeutics Co., Ltd	2023.3	NCT05776355
	TROP2	M.D. Anderson Cancer Center	2023.6	NCT05922930
	Claudin6	Second Affiliated Hospital of Guangzhou Medical University	2022.6	NCT05410717
Prostate cancer	PSMA	Allife Medical Science and Technology Co., Ltd	2018.1	NCT03692663
Colorectal cancer	NKG2D	Zhejiang University	2021.12	NCT05213195
	MUC1	PersonGen BioTherapeutics (Suzhou) Co., Ltd	2016.7	NCT02839954
ES-SCLC	DLL3	Tianjin Medical University Cancer Institute and Hospital	2022.8	NCT05507593
Hepatocellular carcinoma	MUC1	PersonGen BioTherapeutics (Suzhou) Co., Ltd	2016.7	NCT02839954
	GPC3	Shantou University Medical College	2023.5	NCT05845502
Pancreatic cancer	ROBO1	Asclepius Technology Company Group (Suzhou) Co., Ltd	2019.5	NCT03941457
Pancreatic cancer	NKG2D	Zhejiang University	2024.7	NCT06503497
	NKG2D	Zhejiang University	2024.6	NCT06478459
	Claudin18.2	Zhejiang Provincial People’s Hospital	2024.7	NCT06464965
Testis cancer, refractory endometrial cancer recurrent	Claudin6	Second Affiliated Hospital of Guangzhou Medical University	2022.6	NCT05410717
Advanced renal cell carcinoma, advanced mesothelioma and osteosarcoma	CD70	M.D. Anderson Cancer Center	2023.3	NCT05703854
TNBC	MESO	First Affiliated Hospital of Shantou University Medical College	2023.2	NCT05686720
Head and neck cancer	PD-L1	National Cancer Institute (NCI)	2021.12	NCT04847466
Glioblastoma	HER2	Johann Wolfgang Goethe University Hospital	2017.12	NCT03383978
	MUC1	PersonGen BioTherapeutics (Suzhou) Co., Ltd	2016.7	NCT02839954
Other solid tumors with suitable targets	ROBO1	Asclepius Technology Company Group (Suzhou) Co., Ltd	2019.5	NCT03940820
	NKG2D-Ligand	The Third Affiliated Hospital of Guangzhou Medical University	2018.1	NCT03415100
	ROBO1	Asclepius Technology Company Group (Suzhou) Co., Ltd	2019.5	NCT03931720
	TROP2	M.D. Anderson Cancer Center	2023.1	NCT06066424
	NKG2D	Xinxiang Medical University	2023.1	NCT05528341
	N/A	The Second Hospital of Shandong University	2024.3	NCT06572956
Gastric carcinoma	MUC1	PersonGen BioTherapeutics (Suzhou) Co., Ltd	2016.7	NCT02839954
	PD-L1	National Cancer Institute (NCI)	2021.12	NCT04847466
	Claudin18.2	Zhejiang Provincial People’s Hospital	2024.7	NCT06464965

In addition to targeting tumor cells via CAR-dependent mechanisms, CAR-NK cells can potentially eliminate cancer cells through CAR-independent pathways. NK cells naturally express a wide array of activating receptors, such as natural cytotoxicity receptors (NKp46, NKp44 and NKp30), NKG2D, the costimulatory receptor DNAM-1 (CD226) and certain activating KIRs (KIR2DS1, KIR2DS4 and KIR2DL4) [125, 126]. These receptors enable NK cells to recognize specific ligands expressed on tumor cells and initiate cytotoxicity. Additionally, the expression of CD16 allows NK cells to mediate ADCC, which distinguishes CD16 from other activating receptors by promoting NK cell activation during ADCC [127].

In addition to their role as direct cytotoxic effector cells, activated NK cells secrete various inflammatory cytokines and chemokines, further recruiting and activating other immune cells, such as T cells, dendritic cells and macrophages. This can help overcome the immunosuppressive environment of the TME, facilitating a synergistic effect with other CAR-modified immune cells [27, 122, 128].

As previously mentioned, NK cells offer several advantages over T cells, including diverse sources. NK cells can be isolated from PB, UCB, human embryonic stem cells (hESCs) and iPSCs. Furthermore, NK cell lines such as NK-92 provide a convenient and readily available option for generating CAR-NK cells, meeting the clinical standards required for GMP-compliant CAR-NK product manufacturing [10]. NK cells derived from hESCs and iPSCs, when transfected via non-viral vectors, have demonstrated high transfection efficiency and enhanced CAR expression [129].

CAR-NK therapy presents a promising solution to the challenges that hinder CAR-T cell therapy in solid tumors, but success will depend on strategic innovations. The lower incidence of CRS and GvHD with CAR-NK cells reflects distinct activation mechanisms, positioning them as safer alternatives for patients who cannot tolerate CAR-T cell therapy-related inflammatory risk. However, addressing the poor infiltration of immune cells into solid tumors remains critical. Leveraging insights from HEV biology to engineer CAR-NK cells with enhanced homing capabilities could improve therapeutic outcomes. Additionally, CAR-NK therapies can be further optimized by combining CAR-dependent and CAR-independent cytotoxic pathways, making them versatile tools against heterogeneous tumors. Future efforts should also explore the integration of immune checkpoint inhibitors with CAR-NK cells to increase persistence within the TME and prevent early exhaustion. These strategies will be essential to unlock the full potential of CAR-NK therapies, translating their preclinical promise into durable, real-world outcomes for solid tumor patients.

## Evaluation of the application prospects of CAR-NK cells

Despite promising preclinical findings regarding the efficacy of CAR-NK cells against solid tumors, several challenges must be addressed before their clinical application, such as the following:

### Safety issues

Safety is a critical consideration in the therapeutic use of CAR-modified effector cells. While clinical trial data remain limited, safety concerns such as off-target effects, GvHD, CRS, tumor lysis syndrome and toxicity to normal tissues—observed in CAR-T cell therapy—may also occur in CAR-NK-cell therapy [130]. For example, a case in which a lung cancer patient receiving CAR-NK immunotherapy after resection experienced a cytokine storm and sudden death following the fourth treatment was reported [131]. Owing to the unique properties of CAR-NK agents, transduction via retroviral and lentiviral vectors may also increase the risk of infection. Additionally, the use of IL-15 to increase NK cell proliferation and cytotoxicity has been linked to abnormal NK cell proliferation, potentially leading to leukemia [132]. NK-92 cells, as tumor-derived cells, carry the risk of allogeneic tumorigenicity in clinical applications. Radiation is often applied to reduce CAR-NK cell proliferation before infusion; however, this approach may compromise the effectiveness of CAR-NK cells [19]. Compared with that of CAR-T cells, the safety of CAR-NK cells has improved. Because of immune system tightness and complexity, there may still be different safety problems, such as severe inflammatory reactions caused by excessive immune responses and unpredictable results after genetic modification, such as leukemia tendency. Therefore, establishing comprehensive monitoring systems and emergency protocols is essential to manage adverse events. Furthermore, the addition of a self-destructive mechanism preventing the consequences of overtreatment should also be considered in CAR-NK design.

### Preparation challenges

The preparation process for CAR-NK cells is complex, and the stability of CAR expression can vary depending on the method used. Moreover, mature NK cells have a short lifespan and limited stability in vivo, necessitating in vitro expansion to obtain sufficient numbers of CAR-NK cells for clinical applications. As a result, both the method of introducing CARs into NK cells and the process of NK cell expansion in vitro require further research [2]. Recent studies have shown that the use of non-viral sleeping beauty transposable elements derived from minimal DNA vectors to generate CAR-NK cells results in increased CAR expression and stability while also increasing CAR cytotoxicity to a certain degree [133]. Overall, although there are many sources, the transformation of NK cells into CAR-NK cells is still relatively simple, and the success rate is not ideal. The time-consuming CAR-NK preparation method may have difficulty meeting the needs of clinical patients. There is a need to develop rapid preparation and amplification technology, or to develop different targets, for the clinical treatment of CAR-NK cell lines for shelf-like supplies.

### Increased antitumor effects of CAR-NK cells

In the TME, inhibitory cytokines, coinhibitory receptors expressed by tumor cells (such as PD-1, NKG2A and TIM-3) and their ligands, regulatory T cells (Tregs) and myeloid-derived suppressor cells (MDSCs), are crucial mechanisms of tumor immune escape [134]. These factors also significantly inhibit the activity of CAR-NK cells [6]. In addition to these classical immune mediators, metabolic products such as prostaglandin E2 (PGE₂) and adenosine are abundantly secreted in the TME and have been shown to impair NK cell viability, effector cytokine secretion and cytotoxicity. Acting in concert with cytokine-mediated pathways, these soluble metabolites further exacerbate the functional inhibition of CAR-NK cells in solid tumors [135]**.** Another major barrier is the immunosuppressive role of tumor-derived exosomes (TDEs). These extracellular vesicles carry suppressive cargoes that inhibit NK cell activation, downregulate activating receptors and diminish cytolytic function. For instance, exosomes derived from pancreatic cancer cells have been shown to reduce the expression of NKG2D and NKp30 on NK cells, leading to impaired tumor cell killing. Such exosome-mediated signaling may similarly compromise CAR-NK activity within the hostile TME [136].

To counteract TME-mediated suppression, several engineering strategies have been proposed. For example, CAR expression on iPSC-derived NK cells alone does not elicit robust cytotoxicity against hepatocellular carcinoma (HCC). However, when CAR-NK cells targeting GPC3 or AFP were engineered with TGF-*β* receptor 2 knockout (TGF*β*R2-KO) or a dominant-negative receptor (TGF*β*R2-DN), enhanced antigen-specific cytolytic activity was observed, suggesting that disrupting inhibitory signaling pathways may restore CAR-NK function [137]. In another approach, manganese dioxide (MnOX) nanoparticles with peroxidase-like activity have been utilized to catalyze the decomposition of hydrogen peroxide (H_2_O_2_), alleviating hypoxia in the TME. When functionalized with CD56 antibodies, these nanoparticles selectively bind to CAR-NK cells and significantly improve their cytotoxicity under hypoxic conditions [138].

Direct enhancement of CAR-NK cell cytotoxicity, such as improving CAR signaling, offers a promising strategy to overcome the immunosuppressive environment. A notable example is the addition of costimulatory domains in first- to third-generation CAR structures. For example, a study showed that the CD28 costimulatory domain enhances CAR signal transduction in NK cells via the LCK/CD3Z/ZAP70 signaling axis [139]. The DNAM1 and 2B4 costimulatory domains also improved the cytotoxicity of anti-GPC3 CAR-NK cells in vitro against hepatocellular carcinoma cells [140]. Additionally, DAP10, a transmembrane adaptor protein, forms the KLRK1-HCST-activating receptor, which may further increase CAR-NK cell activity [140].

Beyond receptor-level engineering, recent studies have demonstrated that intrinsic inhibitory molecules also limit CAR-NK cell efficacy. For instance, CISH knockout in CAR-NK cells significantly enhanced in vivo persistence and tumor clearance in preclinical models. Moreover, genome-wide CRISPR screens have identified novel negative regulators such as CALHM2, whose silencing improved anti-tumor activity. These findings suggest that targeting intrinsic NK suppressors represents a promising strategy to optimize CAR-NK functionality [141]**.**

The previously mentioned use of non-viral sleeping beauty transposons from minimal DNA vectors also results in the production of more cytotoxic CAR-NK cells [133].

Fourth-generation CARs introduce an intracellular domain containing small molecules (such as IL-15 [26]), which enhances the antitumor efficacy of CAR-NK cells by promoting NK cell proliferation. This approach also directly boosts the killing ability of CAR-NK cells through the localized production of specific cytokines (e.g., IL-15), allowing them to eliminate cancer cells and cancer-associated fibroblasts, disrupt solid tumor structures and enhance therapeutic effects. It has potential for treating fibroproliferative solid tumors [56]. However, attention should be given to local and systemic toxic reactions that may arise due to cytokine release [142].

Theoretically, the antitumor effect of CAR-NK can be achieved by the front and back sides, namely, weakening the influence of the tumor immunosuppressive microenvironment and enhancing the direct cytotoxicity of CAR-NK. Why can the immunosuppressive factor in the TME be turned into a “catalyst” to activate CAR-NK cells? More complicated studies are needed to systematically compare the degree of CAR-NK function activation and select the corresponding intracellular segment with strong activation ability for combined application.

### On-target/off-tumor effects

Because the antigen targeted by CAR-NK cells is not exclusively expressed on tumors, CAR-NK cells may mistakenly bind to normal cells expressing the same antigen, leading to an “on-target/off-tumor” effect and resulting in accidental killing. Furthermore, during the CAR-NK-mediated killing of tumor cells, receptor–antigen ligation can induce the transfer of target antigens from the surface of tumor cells to CAR-NK cells via endocytosis, resulting in the production of NK cells that carry cytoplasmic antigens (NKTROG +). This process weakens the tumor surface antigen, diminishes the effectiveness of CAR-NK therapy and can lead to tumor recurrence in patients with low antigen expression, hindering subsequent treatments. Simultaneously, CAR-NK cells expressing the target antigen may be attacked by other CAR-NK cells, resulting in a cannibalism-like phenomenon [143]. To avoid this, researchers have developed a dual CAR system that combines an activated CAR (aCAR) targeting a tumor antigen with an inhibitory NK self-recognition CAR (iCAR). When bound to CAR-NK cells, this system delivers a “don’t kill me” signal to NK cells, preventing fetal antigen-mediated cannibalism while preserving aCAR signaling against tumor antigens. Moreover, CAR-NK cell activity is enhanced [139].

To increase the targeting accuracy of CAR-immune cells, SynNotch-CAR technology modifies the Notch receptor and its intracellular transcriptional regulatory region by applying “and,” “or” and “not” logic gates to construct CARs [144, 145]. This allows matching with the ectopic expression of different target antigens. Mammals possess Notch receptors 1–4, which consist of an extracellular segment primarily composed of multiple repeated EGF sequences that bind to ligands such as Delta-like 1, Delta-like 3, Delta-like 4, Jagged 1 and Jagged 2. The intracellular region includes a nuclear localization signal domain, a transcriptional regulation domain and an RAM domain. To achieve tumor-specific targeting of the Notch protein, the extracellular segment of Notch can be modified into a scFv, while the intracellular segment can be replaced with GAL4-VP64, LexA-VP64 or GAL4-KRAB. (Gal4 and LexA are DNA-binding domains, with VP64 and KRAB providing transcriptional regulation functions—activation and inhibition, respectively) [146–149]. This setup can cooperate with the directly imported CAR pathway to construct different logic gates.

The AND gate allows for the simultaneous recognition of multiple proteins, wherein a target cell must express proteins A, B, C, etc., to trigger cell death: “If [target cell expresses A] and [target cell expresses B] and [target cell expresses C], then [kill target cells].”

The OFF gate, or simple OFF gate, targets antigens that are expressed on normal cells but not on cancer cells. This is achieved by incorporating an inhibitory CAR (iCAR) to construct a transcriptional activation domain capable of activating apoptotic proteins as the intracellular component of the synNotch-CAR. Among the apoptotic proteins tested, tBID has proven to be the most effective.

**Fig. 2:**
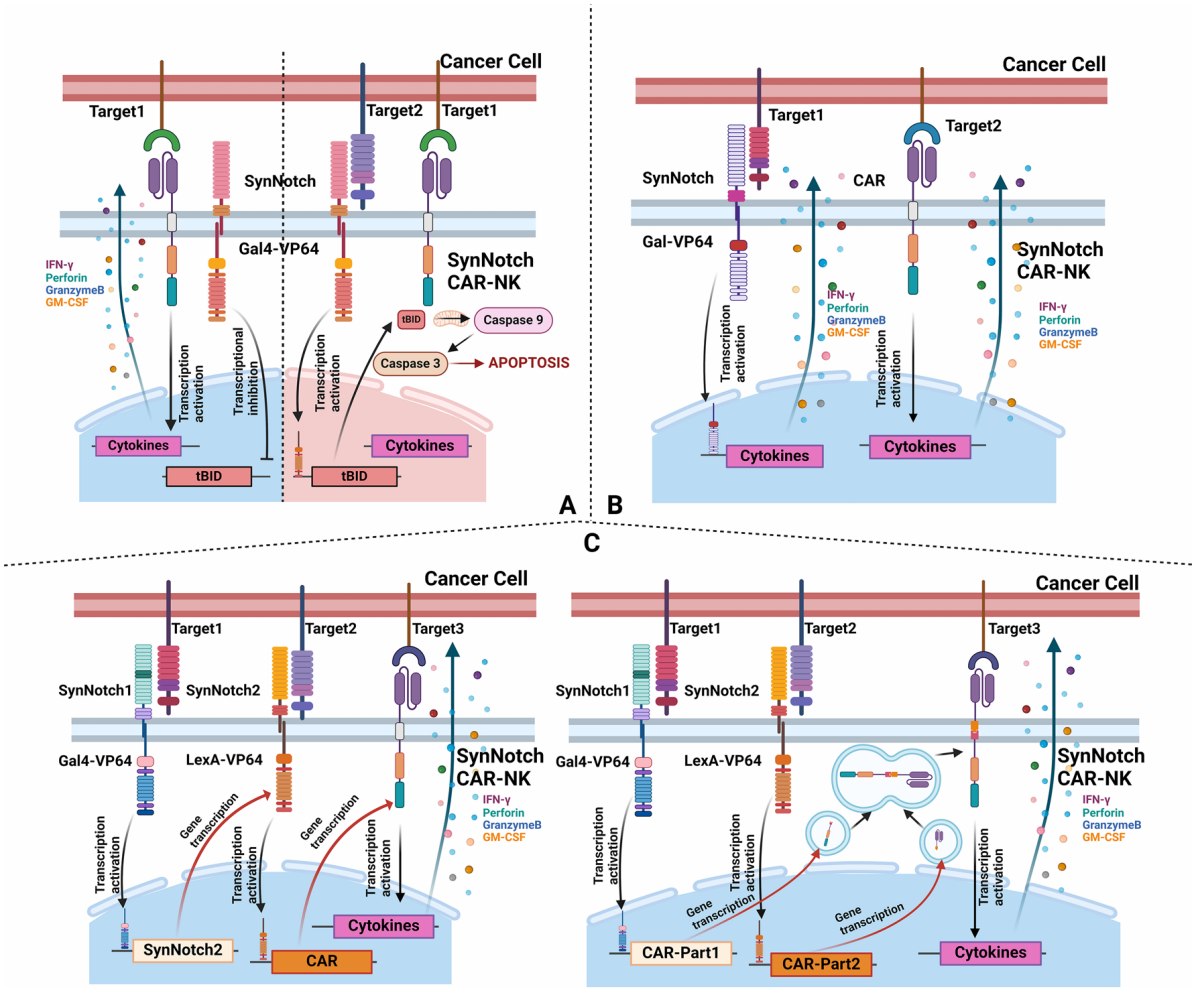
Schematic representation of the three SynNotch-CAR-NK logic gates **A** OFF gate; **B** OR gate; **C** AND gate, including two methods: parallel (left) and series (right). This figure was drawn via Biorender

The OR gate does not inherently require synNotch; it can be implemented simply by constructing two or more targeted CARs. However, the OR gate can be combined with other logic gates. For example, if { [there is A on the surface of the target cell] or [there is B on the surface of the target cell]} and [there is C on the surface of the target cell], then [kill the target cell]. If { [target cell has A] and [target cell has B]} or [target cell has C], then [kill the target cell]. A schematic diagram of the above logic gate is shown in Fig. 2.

These logic gates were initially applied in the construction of CAR-T cells [145, 150–152]. Researchers have since attempted to adapt this technology for CAR-NK cells by constructing HER2 synNotch/CEA-CAR-NK cells. Through the implementation of the AND gate, they successfully targeted and killed colorectal cancer cells that highly expressed HER2 and CEA while sparing normal cells with physiological levels of HER2 expression [153].

Because tumor-specific markers have not yet been identified completely, CAR-NK cells need to be designed to identify the target more accurately so as not to cause “injury.” The logic door can provide multiple “identifications,” allowing CAR-NK cells to more accurately recognize tumors. More work is needed to identify more accurate, more specific tumor markers in the future.

### Combination strategies with CAR-NK cells

Given the complex and immunosuppressive nature of the TME, combining CAR-NK cell therapy with other therapeutic modalities has become a promising strategy to enhance treatment efficacy in solid tumors [154, 155]. Immune checkpoint inhibitors (ICIs), such as PD-1/PD-L1 or CTLA-4 blockers, have demonstrated synergistic effects when combined with adoptive cell therapies. ICIs can relieve the suppression of endogenous and adoptively transferred immune cells, thus improving CAR-NK cell persistence and antitumor activity. Preclinical studies have shown that the combination of CAR-NK cells and ICIs promotes tumor regression and prolongs survival in murine models of melanoma and glioblastoma [156].

In addition to immunomodulatory agents, chemotherapeutic and targeted drugs can also enhance the function of CAR-NK cells. Chemotherapy can increase the immunogenicity of tumor cells by inducing immunogenic cell death and upregulating stress-induced ligands, making tumor cells more susceptible to NK cell-mediated killing. Targeted therapies, such as tyrosine kinase inhibitors, can remodel the TME by reducing suppressive cell populations, thus facilitating the infiltration and activity of CAR-NK cells. Moreover, some agents have been shown to directly upregulate activating ligands for NK cells on tumor targets, further potentiating CAR-NK cytotoxicity [157].

In addition to systemic therapeutic combinations, regional therapies have emerged as promising adjuncts to enhance CAR-NK activity in solid tumors. Among them, mild photothermal therapy (PTT) can increase tumor perfusion, promote immunogenic cell death, and facilitate immune cell infiltration by triggering the release of tumor antigens and danger-associated molecular patterns (DAMPs). In preclinical lung cancer models, the combination of PTT and CAR-NK therapy led to complete eradication of residual tumor masses, highlighting the synergistic potential of this strategy. Such local interventions may effectively convert immunologically “cold” tumors into “hot” tumors, thereby enhancing CAR-NK therapeutic efficacy in otherwise non-responsive tumor types [139]**.**

Collectively, these combination strategies—whether systemic or localized—offer multiple avenues to overcome the physical and immunological barriers of solid tumors. Future efforts should focus on optimizing the timing, dosage and sequencing of CAR-NK therapy with adjunctive treatments to maximize therapeutic synergy while minimizing adverse effects.

Recent advances in synthetic receptor engineering provide additional synergistic strategies. SynNotch-based circuits allow for precise control of CAR-NK cell activation in response to specific TME cues. For example, Bender et al. developed a synNotch-TIGIT receptor that senses CD155 on glioblastoma cells and drives the secretion of anti-CD73 scFv, thereby neutralizing local adenosine-mediated immunosuppression and improving cytotoxic efficacy [158]. Similarly, Ahmadnia et al. engineered a PD1-synNotch receptor in NK cells that triggers IL-12 production upon engagement with PDL1 + tumor cells, enhancing IFN-γ release and antitumor activity [159]. These approaches exemplify how integrating receptor logic circuits with cytokine modulation can reshape the TME and amplify CAR-NK therapeutic responses in a highly controlled manner.

Collectively, these combination strategies—whether systemic, localized, or receptor-engineered—offer diverse avenues to overcome the immunologic and physical barriers of solid tumors. Future efforts should focus on optimizing timing, dosing, and delivery to fully realize the synergistic potential of CAR-NK-based combination therapies.

## Conclusions and prospects

This article provides a brief overview of the preparation methods for CAR-NK cells and reviews recent research progress in CAR-NK therapy for tumors, with a primary focus on preclinical studies and the underlying antitumor mechanisms. CAR-NK cells have gradually emerged as a promising area in tumor immunotherapy, demonstrating a lower likelihood of serious adverse reactions such as CRS, suggesting a favorable research trajectory. Furthermore, the ability of NK cells to mediate ADCC and recruit other immune cells opens the door for potentially fruitful combinations of monoclonal antibodies and CAR-T cell immunotherapy. The evolution from CAR-T cells to CAR-NK cells indicates that different immune cells can harness their unique antitumor effects through CAR technology. For example, CAR-M cells have also entered clinical trials (NCT06224738), and the innovative design of their intracellular segments offers new ideas for developing CAR-M cells and even CAR-DCs (dendritic cells). Although preclinical studies have shown that CAR-NK therapy is a promising modality for treating solid tumors, the immunosuppressive TME remains a significant barrier to the sustained efficacy of this therapy. Enhancing CAR-NK cell persistence, possibly through genetic modifications or cytokine support systems, will be critical to maintain therapeutic efficacy over time. Moreover, delicate studies that integrate CAR-NK therapy with immune checkpoint inhibitors or other systemic treatments are needed to increase the effectiveness of CAR-NK therapy in the future. In addition to tumor immunotherapy, CAR-NK cells have demonstrated excellent efficacy in other areas, including SARS-CoV-2 [160] and anti-HIV [161] immunotherapy. The insights gained from the research summarized above can provide strategic support for the development of the next generation of CAR technology and inspire broader applications of CAR technology across various fields.

## Supplementary Information

Below is the link to the electronic supplementary material.

Supplementary file1 (DOCX 666 kb)

## Data Availability

No datasets were generated or analyzed during the current study.

## Abbreviations

ADCC: Antibody-dependent cell-mediated cytotoxicity
ADT: Androgen deprivation therapy
AR: Androgen receptor
B-ALL: Acute B-cell lymphoblastic leukemia
B-NHL: B-cell non-Hodgkin’s lymphoma
BC: Breast cancer
CAFs: Cancer-associated fibroblasts
CAR: Chimeric antigen receptor
CAR-T: Chimeric antigen receptor-T
CAR-NK: Chimeric antigen receptor-NK
CRC: Colorectal cancer
CEA: Carcinoembryonic antigen
CHT: Caudal hematopoietic tissue
CRS: Cytokine release syndrome
CRP: C-reactive protein
CLS: Capillary leak syndrome
CRPC: Castration-resistant PCa
DIC: Disseminated intravascular coagulation
DIPG: Diffuse intrinsic pontine glioma
DLL3: Delta-like ligand 3
DR4: Death receptor 4
DMSO: Dimethyl sulfoxide
EBV-LCL: Epstein‒Barr lymphoblastoid cell line
EpCAM: Epithelial cell adhesion molecule
EGFR: Epidermal growth factor receptor
ER: Estrogen receptor
ESCC: Esophageal squamous cell carcinoma
FR*α*: Folate receptor alpha
GBM: Glioblastoma
GD2: Disialoganglioside GD2
GPC3: Glypican-3
HCC: Hepatocellular carcinoma
HEVs: High endothelial venules
HER2: Human epidermal growth factor receptor 2
hESCs: Human embryonic stem cells
IPSCs: Induced pluripotent stem cells
ICANS: Immune effector cell-associated neurotoxicity syndrome
IL: Interleukin
ITAMS: Immunoreceptor tyrosine-based activation motifs
LUAD: Lung adenocarcinoma
mbIL2: Membrane-bound IL-2
mbIL13: Membrane-bound IL-13
c-MET: Mesenchymal epithelial transition factor
MSLN: Mesothelin
MCTS: Multicellular tumor spheroid
MDSCs: Myeloid-derived suppressor cells
MnOX: Manganese dioxide
MLVs: Murine leukemia viruses
NK: Natural killer
NSCLC: Non-small cell lung cancer
PB: Peripheral blood
PBNK: Peripheral blood natural killer
PC: Pancreatic cancer
PCa: Prostate cancer
PR: Progesterone receptor
PDAC: Pancreatic ductal adenocarcinoma
PSMA: Prostate-specific membrane antigen
PSO: Patient-specific organoid
PSCA: Prostate stem cell antigen
OC: Ovarian cancer
OTSCC: Oral tongue squamous cell carcinoma
scFv: Single-chain antibody fragment
SCC: Squamous cell carcinoma
SCLC: Small cell lung cancer
SLAM: Signaling lymphocytic activation molecule
STING: Stimulator of interferon genes
TAAs: Tumor-associated antigens
TF: Tissue factor
TGF-β: Transforming growth factor β
TME: Tumor microenvironment
TNBC: Triple-negative breast cancer
TNFR: Tumor necrosis factor receptor
UCB: Umbilical cord blood
VL: Variable light chain
VH: Variable heavy chain

## References

[1] Stoiber S, Cadilha BL, Benmebarek MR et al (2019) Limitations in the design of chimeric antigen receptors for cancer therapy. Cells. 10.3390/cells8050472 31108883 10.3390/cells8050472 PMC6562702

[2] Gong Y, Klein Wolterink RGJ, Wang J et al (2021) Chimeric antigen receptor natural killer (CAR-NK) cell design and engineering for cancer therapy. J Hematol Oncol 14(1):73. 10.1186/s13045-021-01083-5 33933160 10.1186/s13045-021-01083-5 PMC8088725

[3] Liu E, Marin D, Banerjee P et al (2020) Use of CAR-transduced natural killer cells in CD19-positive lymphoid tumors. N Engl J Med 382(6):545–553. 10.1056/NEJMoa1910607 32023374 10.1056/NEJMoa1910607 PMC7101242

[4] Shah NN, Lee DW, Yates B et al (2021) Long-term follow-up of CD19-CAR T-cell therapy in children and young adults with B-ALL. J Clin Oncol 39(15):1650–1659. 10.1200/jco.20.02262 33764809 10.1200/JCO.20.02262 PMC8274806

[5] Zhang J, Hu Y, Yang J et al (2022) Non-viral, specifically targeted CAR-T cells achieve high safety and efficacy in B-NHL. Nature 609(7926):369–374. 10.1038/s41586-022-05140-y 36045296 10.1038/s41586-022-05140-y PMC9452296

[6] Jing J, Ma Y, Xie Z et al (2024) Acute T-cell lymphoblastic leukemia: chimeric antigen receptor technology may offer a new hope. Front Immunol 15:1410519. 10.3389/fimmu.2024.1410519 39192970 10.3389/fimmu.2024.1410519 PMC11347323

[7] Schubert ML, Schmitt M, Wang L et al (2021) Side-effect management of chimeric antigen receptor (CAR) T-cell therapy. Ann Oncol 32(1):34–48. 10.1016/j.annonc.2020.10.478 33098993 10.1016/j.annonc.2020.10.478

[8] Liu S, Galat V, Galat Y et al (2021) NK cell-based cancer immunotherapy: from basic biology to clinical development. J Hematol Oncol 14(1):7. 10.1186/s13045-020-01014-w 33407739 10.1186/s13045-020-01014-w PMC7788999

[9] Herberman RB, Nunn ME, Holden HT et al (1975) Natural cytotoxic reactivity of mouse lymphoid cells against syngeneic and allogeneic tumors. II. Characterization of effector cells. Int J Cancer 16(2):230–239. 10.1002/ijc.2910160205 1080480 10.1002/ijc.2910160205

[10] Xie G, Dong H, Liang Y et al (2020) CAR-NK cells: a promising cellular immunotherapy for cancer. EBioMedicine 59:102975. 10.1016/j.ebiom.2020.102975 32853984 10.1016/j.ebiom.2020.102975 PMC7452675

[11] Del Zotto G, Antonini F, Pesce S et al (2020) Comprehensive phenotyping of human PB NK cells by flow cytometry. Cytometry A 97(9):891–899. 10.1002/cyto.a.24001 32198974 10.1002/cyto.a.24001

[12] Poli A, Michel T, Thérésine M et al (2009) CD56bright natural killer (NK) cells: an important NK cell subset. Immunology 126(4):458–465. 10.1111/j.1365-2567.2008.03027.x 19278419 10.1111/j.1365-2567.2008.03027.x PMC2673358

[13] Ben-Eliyahu S, Page GG, Yirmiya R et al (1999) Evidence that stress and surgical interventions promote tumor development by suppressing natural killer cell activity. Int J Cancer 80(6):880–888. 10.1002/(sici)1097-0215(19990315)80:6%3c880::aid-ijc14%3e3.0.co;2-y 10074922 10.1002/(sici)1097-0215(19990315)80:6<880::aid-ijc14>3.0.co;2-y

[14] Hensler T, Hecker H, Heeg K et al (1997) Distinct mechanisms of immunosuppression as a consequence of major surgery. Infect Immun 65(6):2283–2291. 10.1128/iai.65.6.2283-2291.1997 9169765 10.1128/iai.65.6.2283-2291.1997 PMC175317

[15] Shakhar G, Ben-Eliyahu S (2003) Potential prophylactic measures against postoperative immunosuppression: could they reduce recurrence rates in oncological patients? Ann Surg Oncol 10(8):972–992. 10.1245/aso.2003.02.007 14527919 10.1245/aso.2003.02.007

[16] Merino A, Maakaron J, Bachanova V (2023) Advances in NK cell therapy for hematologic malignancies: NK source, persistence and tumor targeting. Blood Rev 60:101073. 10.1016/j.blre.2023.101073 36959057 10.1016/j.blre.2023.101073 PMC10979648

[17] Miller JS, Soignier Y, Panoskaltsis-Mortari A et al (2005) Successful adoptive transfer and in vivo expansion of human haploidentical NK cells in patients with cancer. Blood 105(8):3051–3057. 10.1182/blood-2004-07-2974 15632206 10.1182/blood-2004-07-2974

[18] Vacca A, Di Stefano R, Frassanito A et al (1991) A disturbance of the IL-2/IL-2 receptor system parallels the activity of multiple myeloma. Clin Exp Immunol 84(3):429–434 2044221 PMC1535424

[19] Klingemann H, Boissel L, Toneguzzo F (2016) Natural killer cells for immunotherapy - advantages of the NK-92 cell line over blood NK cells. Front Immunol 7:91. 10.3389/fimmu.2016.00091 27014270 10.3389/fimmu.2016.00091 PMC4789404

[20] Ayala Ceja M, Khericha M, Harris CM et al (2024) CAR-T cell manufacturing: major process parameters and next-generation strategies. J Exp Med. 10.1084/jem.20230903 38226974 10.1084/jem.20230903 PMC10791545

[21] Ying Z, Huang XF, Xiang X et al (2019) A safe and potent anti-CD19 CAR T cell therapy. Nat Med 25(6):947–953. 10.1038/s41591-019-0421-7 31011207 10.1038/s41591-019-0421-7 PMC7518381

[22] Alabanza L, Pegues M, Geldres C et al (2017) Function of novel anti-CD19 chimeric antigen receptors with human variable regions is affected by hinge and transmembrane domains. J Mole Ther. 25(11):2452–2465 10.1016/j.ymthe.2017.07.013 PMC5675490 28807568

[23] Li Y, Hermanson DL, Moriarity BS et al (2018) Human iPSC-derived natural killer cells engineered with chimeric antigen receptors enhance anti-tumor activity. Cell Stem Cell 23(2):181-192.e5. 10.1016/j.stem.2018.06.002 30082067 10.1016/j.stem.2018.06.002 PMC6084450

[24] Ghorai SK, Pearson AN (2024) Current strategies to improve chimeric antigen receptor T (CAR-T) cell persistence. Cureus 16(7):e65291. 10.7759/cureus.65291 39184661 10.7759/cureus.65291 PMC11343441

[25] MacKay M, Afshinnekoo E, Rub J et al (2020) The therapeutic landscape for cells engineered with chimeric antigen receptors. Nat Biotechnol 38(2):233–244. 10.1038/s41587-019-0329-2 31907405 10.1038/s41587-019-0329-2

[26] Wang X, Jasinski DL, Medina JL et al (2020) Inducible MyD88/CD40 synergizes with IL-15 to enhance antitumor efficacy of CAR-NK cells. Blood Adv 4(9):1950–1964. 10.1182/bloodadvances.2020001510 32384544 10.1182/bloodadvances.2020001510 PMC7218419

[27] Hu Y, Tian ZG, Zhang C (2018) Chimeric antigen receptor (CAR)-transduced natural killer cells in tumor immunotherapy. Acta Pharmacol Sin 39(2):167–176. 10.1038/aps.2017.125 28880014 10.1038/aps.2017.125 PMC5800464

[28] Bexte T, Reindl LM, Ullrich E (2023) Nonviral technologies can pave the way for CAR-NK cell therapy. J Leukoc Biol 114(5):475–486. 10.1093/jleuko/qiad074 37403203 10.1093/jleuko/qiad074

[29] Hacein-Bey-Abina S, Garrigue A, Wang GP et al (2008) Insertional oncogenesis in 4 patients after retrovirus-mediated gene therapy of SCID-X1. J clin investig 118(9):3132–3142 18688285 10.1172/JCI35700 PMC2496963

[30] Zheng Z, Chinnasamy N, Morgan RA (2012) Protein L: a novel reagent for the detection of chimeric antigen receptor (CAR) expression by flow cytometry. J Transl Med 10:29. 10.1186/1479-5876-10-29 22330761 10.1186/1479-5876-10-29 PMC3299624

[31] Schanda N, Sauer T, Kunz A et al (2021) Sensitivity and specificity of CD19.CAR-T cell detection by flow cytometry and PCR. Cells. 10.3390/cells10113208 34831430 10.3390/cells10113208 PMC8621201

[32] Chen L, Liu S, Adah D et al (2023) Soluble programmed death ligand-1-induced immunosuppressive effects on chimeric antigen receptor-natural killer cells targeting Glypican-3 in hepatocellular carcinoma. Immunology 169(2):204–218. 10.1111/imm.13624 36640111 10.1111/imm.13624 PMC10712665

[33] Phan MT, Lee SH, Kim SK et al (2016) Expansion of NK cells using genetically engineered K562 feeder cells. Methods Mol Biol 1441:167–174. 10.1007/978-1-4939-3684-7_14 27177665 10.1007/978-1-4939-3684-7_14

[34] Vidard L, Dureuil C, Baudhuin J et al (2019) CD137 (4–1BB) engagement fine-tunes synergistic IL-15- and IL-21-driven NK cell proliferation. J Immunol 203(3):676–685. 10.4049/jimmunol.1801137 31201235 10.4049/jimmunol.1801137

[35] Ma M, Badeti S, Kim JK et al (2022) Natural Killer (NK) and CAR-NK cell expansion method using membrane bound-IL-21-modified B cell line. J Vis Exp 8(180):10–3791. 10.3791/62336 10.3791/62336 PMC10858653 35225261

[36] Lee DY, Kim Y, Park JS et al (2023) Development of genetically engineered feeder cells for natural killer cell expansion. Anticancer Res 43(9):3897–3904. 10.21873/anticanres.16577 37648291 10.21873/anticanres.16577

[37] Yao X, Matosevic S (2021) Cryopreservation of NK and T cells without DMSO for adoptive cell-based immunotherapy. BioDrugs 35(5):529–545. 10.1007/s40259-021-00494-7 34427899 10.1007/s40259-021-00494-7 PMC12376086

[38] Das S, Niemeyer E, Leung ZA et al (2024) Human natural killer cells cryopreserved without DMSO sustain robust effector responses. Mol Pharm 21(2):651–660. 10.1021/acs.molpharmaceut.3c00798 38230666 10.1021/acs.molpharmaceut.3c00798

[39] Pasley S, Zylberberg C, Matosevic S (2017) Natural killer-92 cells maintain cytotoxic activity after long-term cryopreservation in novel DMSO-free media. Immunol Lett 192:35–41. 10.1016/j.imlet.2017.09.012 28966059 10.1016/j.imlet.2017.09.012

[40] Xu R, Shi X, Huang H et al (2024) Development of a Me(2)SO-free cryopreservation medium and its long-term cryoprotection on the CAR-NK cells. Cryobiology 114:104835. 10.1016/j.cryobiol.2023.104835 38070820 10.1016/j.cryobiol.2023.104835

[41] Berjis A, Muthumani D, Aguilar OA et al (2024) Pretreatment with IL-15 and IL-18 rescues natural killer cells from granzyme B-mediated apoptosis after cryopreservation. Nat Commun 15(1):3937. 10.1038/s41467-024-47574-0 38729924 10.1038/s41467-024-47574-0 PMC11087472

[42] Siegel RL, Giaquinto AN, Jemal A (2024) Cancer statistics, 2024. CA Cancer J Clin 74(1):12–49. 10.3322/caac.21820 38230766 10.3322/caac.21820

[43] Le N, Sund M, Vinci A (2016) Prognostic and predictive markers in pancreatic adenocarcinoma. Dig Liver Dis 48(3):223–230. 10.1016/j.dld.2015.11.001 26769569 10.1016/j.dld.2015.11.001

[44] Teng KY, Mansour AG, Zhu Z et al (2022) Off-the-shelf prostate stem cell antigen-directed chimeric antigen receptor natural killer cell therapy to treat pancreatic cancer. Gastroenterology 162(4):1319–1333. 10.1053/j.gastro.2021.12.281 34999097 10.1053/j.gastro.2021.12.281 PMC8963130

[45] Zuo S, Wen Y, Panha H et al (2017) Modification of cytokine-induced killer cells with folate receptor alpha (FR*α*)-specific chimeric antigen receptors enhances their antitumor immunity toward FR*α*-positive ovarian cancers. Mol Immunol 85:293–304. 10.1016/j.molimm.2017.03.017 28360017 10.1016/j.molimm.2017.03.017

[46] Lee YE, Ju A, Choi HW et al (2020) Rationally designed redirection of natural killer cells anchoring a cytotoxic ligand for pancreatic cancer treatment. J Control Release 326:310–323. 10.1016/j.jconrel.2020.07.016 32682905 10.1016/j.jconrel.2020.07.016

[47] Shah Z, Tian L, Li Z et al (2024) Human anti-PSCA CAR macrophages possess potent antitumor activity against pancreatic cancer. Cell Stem Cell 31(6):803-817.e6. 10.1016/j.stem.2024.03.018 38663406 10.1016/j.stem.2024.03.018 PMC11162318

[48] Adusumilli PS, Zauderer MG, Rivière I et al (2021) A phase I trial of regional mesothelin-targeted CAR T-cell therapy in patients with malignant pleural disease, in combination with the anti-PD-1 agent pembrolizumab. Cancer Discov 11(11):2748–2763. 10.1158/2159-8290.Cd-21-0407 34266984 10.1158/2159-8290.CD-21-0407 PMC8563385

[49] Haas AR, Tanyi JL, O’Hara MH et al (2019) Phase I study of lentiviral-transduced chimeric antigen receptor-modified T cells recognizing mesothelin in advanced solid cancers. Mol Ther 27(11):1919–1929. 10.1016/j.ymthe.2019.07.015 31420241 10.1016/j.ymthe.2019.07.015 PMC6838875

[50] Morello A, Sadelain M, Adusumilli PS (2016) Mesothelin-targeted CARs: Driving T cells to solid tumors. Cancer Discov 6(2):133–146. 10.1158/2159-8290.Cd-15-0583 26503962 10.1158/2159-8290.CD-15-0583 PMC4744527

[51] Zhang Z, Jiang D, Yang H et al (2019) Modified CAR T cells targeting membrane-proximal epitope of mesothelin enhances the antitumor function against large solid tumor. Cell Death Dis 10(7):476. 10.1038/s41419-019-1711-1 31209210 10.1038/s41419-019-1711-1 PMC6572851

[52] Da Y, Liu Y, Hu Y et al (2022) STING agonist cGAMP enhances anti-tumor activity of CAR-NK cells against pancreatic cancer. Oncoimmunology 11(1):2054105. 10.1080/2162402x.2022.2054105 35371622 10.1080/2162402X.2022.2054105 PMC8967397

[53] Flieswasser T, Camara-Clayette V, Danu A et al (2019) Screening a broad range of solid and haematological tumour types for CD70 expression using a uniform IHC methodology as potential patient stratification method. Cancers (Basel). 10.3390/cancers11101611 31652572 10.3390/cancers11101611 PMC6826714

[54] Kong F, Ye Q, Xiong Y (2023) Comprehensive analysis of prognosis and immune function of CD70-CD27 signaling axis in pan-cancer. Funct Integr Genomics 23(1):48. 10.1007/s10142-023-00977-6 36700974 10.1007/s10142-023-00977-6

[55] Yang M, Tang X, Zhang Z et al (2020) Tandem CAR-T cells targeting CD70 and B7–H3 exhibit potent preclinical activity against multiple solid tumors. Theranostics 10(17):7622–7634. 10.7150/thno.43991 32685008 10.7150/thno.43991 PMC7359081

[56] Van den Eynde A, Gehrcken L, Verhezen T et al (2024) IL-15-secreting CAR natural killer cells directed toward the pan-cancer target CD70 eliminate both cancer cells and cancer-associated fibroblasts. J Hematol Oncol 17(1):8. 10.1186/s13045-024-01525-w 38331849 10.1186/s13045-024-01525-w PMC10854128

[57] Barzaman K, Karami J, Zarei Z et al (2020) Breast cancer: biology, biomarkers, and treatments. Int Immunopharmacol 84:106535. 10.1016/j.intimp.2020.106535 32361569 10.1016/j.intimp.2020.106535

[58] Mercogliano MF, Bruni S, Mauro FL et al (2023) Emerging targeted therapies for HER2-positive breast cancer. Cancers (Basel). 10.3390/cancers15071987 37046648 10.3390/cancers15071987 PMC10093019

[59] Musgrove EA, Sutherland RL (2009) Biological determinants of endocrine resistance in breast cancer. Nat Rev Cancer 9(9):631–643. 10.1038/nrc2713 19701242 10.1038/nrc2713

[60] Soleja M, Raj GV, Unni N (2019) An evaluation of fulvestrant for the treatment of metastatic breast cancer. Expert Opin Pharmacother 20(15):1819–1829. 10.1080/14656566.2019.1651293 31486688 10.1080/14656566.2019.1651293

[61] Beeram M, Tan QT, Tekmal RR et al (2007) Akt-induced endocrine therapy resistance is reversed by inhibition of mTOR signaling. Ann Oncol 18(8):1323–1328. 10.1093/annonc/mdm170 17693645 10.1093/annonc/mdm170

[62] Jeselsohn R, Yelensky R, Buchwalter G et al (2014) Emergence of constitutively active estrogen receptor-*α* mutations in pretreated advanced estrogen receptor-positive breast cancer. Clin Cancer Res 20(7):1757–1767. 10.1158/1078-0432.Ccr-13-2332 24398047 10.1158/1078-0432.CCR-13-2332 PMC3998833

[63] Zhang Y, Moerkens M, Ramaiahgari S et al (2011) Elevated insulin-like growth factor 1 receptor signaling induces antiestrogen resistance through the MAPK/ERK and PI3K/Akt signaling routes. Breast Cancer Res 13(3):R52. 10.1186/bcr2883 21595894 10.1186/bcr2883 PMC3218939

[64] Barrow AD, Martin CJ, Colonna M (2019) The natural cytotoxicity receptors in health and disease. Front Immunol 10:909. 10.3389/fimmu.2019.00909 31134055 10.3389/fimmu.2019.00909 PMC6514059

[65] Lin YZ, Lee CC, Cho DY et al (2021) Suppression of breast cancer cells resistant to a pure anti-estrogen with CAR-transduced natural killer cells. Am J Cancer Res 11(9):4455–4469 34659898 PMC8493389

[66] Raftery MJ, Franzén AS, Radecke C et al (2023) Next generation CD44v6-specific CAR-NK cells effective against triple negative breast cancer. Int J Mol Sci. 10.3390/ijms24109038 37240385 10.3390/ijms24109038 PMC10218876

[67] Hu Z (2020) Tissue factor as a new target for CAR-NK cell immunotherapy of triple-negative breast cancer. Sci Rep 10(1):2815. 10.1038/s41598-020-59736-3 32071339 10.1038/s41598-020-59736-3 PMC7028910

[68] Murali Shankar N, Ortiz-Montero P, Kurzyukova A et al (2023) Preclinical assessment of CAR-NK cell-mediated killing efficacy and pharmacokinetics in a rapid zebrafish xenograft model of metastatic breast cancer. Front Immunol 14:1254821. 10.3389/fimmu.2023.1254821 37885894 10.3389/fimmu.2023.1254821 PMC10599014

[69] Kurebayashi J, Okubo S, Yamamoto Y et al (2004) Inhibition of HER1 signaling pathway enhances antitumor effect of endocrine therapy in breast cancer. Breast Cancer 11(1):38–41. 10.1007/bf02968000 14718791 10.1007/BF02968000

[70] Liu Y, Chen J, Tian J et al (2024) Engineered CAR-NK cells with tolerance to H_2_O_2_ and hypoxia can suppress postoperative relapse of triple-negative breast cancers. Cancer Immunol Res. 10.1158/2326-6066.Cir-23-1017 39023168 10.1158/2326-6066.CIR-23-1017

[71] Khan F, Pang L, Dunterman M et al (2023) Macrophages and microglia in glioblastoma: heterogeneity, plasticity, and therapy. J Clin Invest. 10.1172/jci163446 36594466 10.1172/JCI163446 PMC9797335

[72] Brandao M, Simon T, Critchley G et al (2019) Astrocytes, the rising stars of the glioblastoma microenvironment. Glia 67(5):779–790. 10.1002/glia.23520 30240060 10.1002/glia.23520

[73] Han J, Chu J, Chan WK et al (2015) CAR-engineered NK cells targeting wild-type EGFR and EGFRvIII enhance killing of glioblastoma and patient-derived glioblastoma stem cells. Sci Rep 5:11483. 10.1038/srep11483 26155832 10.1038/srep11483 PMC4496728

[74] Jena B, Dotti G, Cooper LJ (2010) Redirecting T-cell specificity by introducing a tumor-specific chimeric antigen receptor. Blood 116(7):1035–1044. 10.1182/blood-2010-01-043737 20439624 10.1182/blood-2010-01-043737 PMC2938125

[75] Sampson JH, Choi BD, Sanchez-Perez L et al (2014) EGFRvIII mCAR-modified T-cell therapy cures mice with established intracerebral glioma and generates host immunity against tumor-antigen loss. Clin Cancer Res 20(4):972–984. 10.1158/1078-0432.Ccr-13-0709 24352643 10.1158/1078-0432.CCR-13-0709 PMC3943170

[76] Müller N, Michen S, Tietze S et al (2015) Engineering NK cells modified with an EGFRvIII-specific chimeric antigen receptor to overexpress CXCR4 improves immunotherapy of CXCL12/SDF-1*α*-secreting glioblastoma. J Immunother 38(5):197–210. 10.1097/cji.0000000000000082 25962108 10.1097/CJI.0000000000000082 PMC4428685

[77] Zhang C, Burger MC, Jennewein L et al (2016) ErbB2/HER2-specific NK cells for targeted therapy of glioblastoma. J Natl Cancer Inst 108(5):djv375. 10.1093/jnci/djv375 10.1093/jnci/djv375 26640245

[78] Burger MC, Forster MT, Romanski A et al (2023) Intracranial injection of natural killer cells engineered with a HER2-targeted chimeric antigen receptor in patients with recurrent glioblastoma. Neuro Oncol 25(11):2058–2071. 10.1093/neuonc/noad087 37148198 10.1093/neuonc/noad087 PMC10628939

[79] Yu AL, Gilman AL, Ozkaynak MF et al (2010) Anti-GD2 antibody with GM-CSF, interleukin-2, and isotretinoin for neuroblastoma. N Engl J Med 363(14):1324–1334. 10.1056/NEJMoa0911123 20879881 10.1056/NEJMoa0911123 PMC3086629

[80] Pule MA, Savoldo B, Myers GD et al (2008) Virus-specific T cells engineered to coexpress tumor-specific receptors: persistence and antitumor activity in individuals with neuroblastoma. Nat Med 14(11):1264–1270. 10.1038/nm.1882 18978797 10.1038/nm.1882 PMC2749734

[81] Zhang J, Webster S, Duffin B et al (2023) Generation of anti-GD2 CAR macrophages from human pluripotent stem cells for cancer immunotherapies. Stem Cell Reports 18(2):585–596. 10.1016/j.stemcr.2022.12.012 36638788 10.1016/j.stemcr.2022.12.012 PMC9968983

[82] Zuo P, Li Y, He C et al (2023) Anti-tumor efficacy of anti-GD2 CAR NK-92 cells in diffuse intrinsic pontine gliomas. Front Immunol 14:1145706. 10.3389/fimmu.2023.1145706 37251413 10.3389/fimmu.2023.1145706 PMC10213244

[83] Vaughan S, Coward JI, Bast RC Jr et al (2011) Rethinking ovarian cancer: recommendations for improving outcomes. Nat Rev Cancer 11(10):719–725. 10.1038/nrc3144 21941283 10.1038/nrc3144 PMC3380637

[84] Hou Y, Zhao X, Nie X (2024) Enhancing the therapeutic efficacy of NK cells in the treatment of ovarian cancer (Review). Oncol Rep. 10.3892/or.2024.8709 38299257 10.3892/or.2024.8709 PMC10851334

[85] Cao B, Liu M, Wang L et al (2020) Use of chimeric antigen receptor NK-92 cells to target mesothelin in ovarian cancer. Biochem Biophys Res Commun 524(1):96–102. 10.1016/j.bbrc.2020.01.053 31980173 10.1016/j.bbrc.2020.01.053

[86] Klapdor R, Wang S, Morgan M et al (2019) Characterization of a novel third-generation anti-CD24-CAR against ovarian cancer. Int J Mol Sci. 10.3390/ijms20030660 30717444 10.3390/ijms20030660 PMC6387114

[87] Ao X, Yang Y, Li W et al (2019) Anti-*α*FR CAR-engineered NK-92 cells display potent cytotoxicity against *α*FR-positive ovarian cancer. J Immunother 42(8):284–296. 10.1097/cji.0000000000000286 31261167 10.1097/CJI.0000000000000286 PMC6735933

[88] Hu T, Li Z, Gao CY et al (2016) Mechanisms of drug resistance in colon cancer and its therapeutic strategies. World J Gastroenterol 22(30):6876–6889. 10.3748/wjg.v22.i30.6876 27570424 10.3748/wjg.v22.i30.6876 PMC4974586

[89] Ang WX, Li Z, Chi Z et al (2017) Intraperitoneal immunotherapy with T cells stably and transiently expressing anti-EpCAM CAR in xenograft models of peritoneal carcinomatosis. Oncotarget 8(8):13545–13559. 10.18632/oncotarget.14592 28088790 10.18632/oncotarget.14592 PMC5355119

[90] Zhang Q, Zhang H, Ding J et al (2018) Combination therapy with EpCAM-CAR-NK-92 cells and regorafenib against human colorectal cancer models. J Immunol Res 2018:4263520. 10.1155/2018/4263520 30410941 10.1155/2018/4263520 PMC6205314

[91] Shiozawa M, Chang CH, Huang YC et al (2018) Pharmacologically upregulated carcinoembryonic antigen-expression enhances the cytolytic activity of genetically-modified chimeric antigen receptor NK-92MI against colorectal cancer cells. BMC Immunol 19(1):27. 10.1186/s12865-018-0262-z 30075754 10.1186/s12865-018-0262-z PMC6091054

[92] Franzén AS, Boulifa A, Radecke C et al (2024) Next-generation CEA-CAR-NK-92 cells against solid tumors: overcoming tumor microenvironment challenges in colorectal cancer. Cancers (Basel). 10.3390/cancers16020388 38254876 10.3390/cancers16020388 PMC10814835

[93] Ruiz-Cordero R, Devine WP (2020) Targeted therapy and checkpoint immunotherapy in lung cancer. Surg Pathol Clin 13(1):17–33. 10.1016/j.path.2019.11.002 32005431 10.1016/j.path.2019.11.002

[94] Liu M, Huang W, Guo Y et al (2022) CAR NK-92 cells targeting DLL3 kill effectively small cell lung cancer cells in vitro and in vivo. J Leukoc Biol 112(4):901–911. 10.1002/jlb.5ma0122-467r 35088475 10.1002/JLB.5MA0122-467R

[95] Peng Y, Zhang W, Chen Y et al (2023) Engineering c-Met-CAR NK-92 cells as a promising therapeutic candidate for lung adenocarcinoma. Pharmacol Res 188:106656. 10.1016/j.phrs.2023.106656 36640859 10.1016/j.phrs.2023.106656

[96] Zhang Y, Zhang C, He M et al (2024) Co-expression of IL-21-enhanced NKG2D CAR-NK cell therapy for lung cancer. BMC Cancer 24(1):119. 10.1186/s12885-023-11806-1 38263004 10.1186/s12885-023-11806-1 PMC10807083

[97] Maluccio M, Covey A (2012) Recent progress in understanding, diagnosing, and treating hepatocellular carcinoma. CA Cancer J Clin 62(6):394–399. 10.3322/caac.21161 23070690 10.3322/caac.21161

[98] Vivier E, Tomasello E, Baratin M et al (2008) Functions of natural killer cells. Nat Immunol 9(5):503–510. 10.1038/ni1582 18425107 10.1038/ni1582

[99] Gao H, Li K, Tu H et al (2014) Development of T cells redirected to glypican-3 for the treatment of hepatocellular carcinoma. Clin Cancer Res 20(24):6418–6428. 10.1158/1078-0432.Ccr-14-1170 25320357 10.1158/1078-0432.CCR-14-1170

[100] Yu M, Luo H, Fan M et al (2018) Development of GPC3-specific chimeric antigen receptor-engineered natural killer cells for the treatment of hepatocellular carcinoma. Mol Ther 26(2):366–378. 10.1016/j.ymthe.2017.12.012 29339014 10.1016/j.ymthe.2017.12.012 PMC5835122

[101] Tseng HC, Xiong W, Badeti S et al (2020) Efficacy of anti-CD147 chimeric antigen receptors targeting hepatocellular carcinoma. Nat Commun 11(1):4810. 10.1038/s41467-020-18444-2 32968061 10.1038/s41467-020-18444-2 PMC7511348

[102] Badeti S, Jiang Q, Naghizadeh A et al (2022) Development of a novel human CD147 knock-in NSG mouse model to test SARS-CoV-2 viral infection. Cell Biosci 12(1):88. 10.1186/s13578-022-00822-6 35690792 10.1186/s13578-022-00822-6 PMC9187929

[103] Sabha Y, Kim SH, Tseng HC et al (2025) CD147-CAR-NK cell therapy shows minimal toxicities in human CD147 transgenic mouse model with solid tumors. Mol Ther Oncolytics 33(1):200957. 10.1016/j.omton.2025.200957 10.1016/j.omton.2025.200957 PMC11952776 40160933

[104] Sawant M, Mahajan K, Renganathan A et al (2022) Chronologically modified androgen receptor in recurrent castration-resistant prostate cancer and its therapeutic targeting. Sci Transl Med 14(649):eabg4132. 10.1126/scitranslmed.abg4132 35704598 10.1126/scitranslmed.abg4132 PMC10259236

[105] Montagner IM, Penna A, Fracasso G et al (2020) Anti-PSMA CAR-engineered NK-92 cells: an off-the-shelf cell therapy for prostate cancer. Cells. 10.3390/cells9061382 32498368 10.3390/cells9061382 PMC7349573

[106] Corchado-Cobos R, García-Sancha N, González-Sarmiento R et al (2020) Cutaneous squamous cell carcinoma: from biology to therapy. Int J Mol Sci. 10.3390/ijms21082956 32331425 10.3390/ijms21082956 PMC7216042

[107] Waldman A, Schmults C (2019) Cutaneous squamous cell carcinoma. Hematol Oncol Clin North Am 33(1):1–12. 10.1016/j.hoc.2018.08.001 30497667 10.1016/j.hoc.2018.08.001

[108] Lin X, Guan T, Xu Y et al (2024) Efficacy of the induced pluripotent stem cell derived and engineered CD276-targeted CAR-NK cells against human esophageal squamous cell carcinoma. Front Immunol 15:1337489. 10.3389/fimmu.2024.1337489 38566988 10.3389/fimmu.2024.1337489 PMC10985341

[109] Liu Q, Cui X, Yu X et al (2017) Cripto-1 acts as a functional marker of cancer stem-like cells and predicts prognosis of the patients in esophageal squamous cell carcinoma. Mol Cancer 16(1):81. 10.1186/s12943-017-0650-7 28431580 10.1186/s12943-017-0650-7 PMC5399850

[110] Liu T, Dai X, Xu Y et al (2023) CD22 is a potential target of CAR-NK cell therapy for esophageal squamous cell carcinoma. J Transl Med 21(1):710. 10.1186/s12967-023-04409-8 37817249 10.1186/s12967-023-04409-8 PMC10563326

[111] Lin X, Guan T, Li Y et al (2024) Efficacy of MUC1-targeted CAR-NK cells against human tongue squamous cell carcinoma. Front Immunol 15:1337557. 10.3389/fimmu.2024.1337557 38390321 10.3389/fimmu.2024.1337557 PMC10882221

[112] Martinez M, Moon EK (2019) CAR T cells for solid tumors: new strategies for finding, infiltrating, and surviving in the tumor microenvironment. Front Immunol 10:128. 10.3389/fimmu.2019.00128 30804938 10.3389/fimmu.2019.00128 PMC6370640

[113] Ager A (2017) High endothelial venules and other blood vessels: critical regulators of lymphoid organ development and function. Front Immunol 8:45. 10.3389/fimmu.2017.00045 28217126 10.3389/fimmu.2017.00045 PMC5289948

[114] Freyer CW, Porter DL (2020) Cytokine release syndrome and neurotoxicity following CAR T-cell therapy for hematologic malignancies. J Allergy Clin Immunol 146(5):940–948. 10.1016/j.jaci.2020.07.025 32771558 10.1016/j.jaci.2020.07.025

[115] Brudno JN, Kochenderfer JN (2016) Toxicities of chimeric antigen receptor T cells: recognition and management. Blood 127(26):3321–3330. 10.1182/blood-2016-04-703751 27207799 10.1182/blood-2016-04-703751 PMC4929924

[116] Cosenza M, Sacchi S, Pozzi S (2021) Cytokine release syndrome associated with T-cell-based therapies for hematological malignancies: pathophysiology, clinical presentation, and treatment. Int J Mol Sci. 10.3390/ijms22147652 34299273 10.3390/ijms22147652 PMC8305850

[117] Lee DW, Kochenderfer JN, Stetler-Stevenson M et al (2015) T cells expressing CD19 chimeric antigen receptors for acute lymphoblastic leukaemia in children and young adults: a phase 1 dose-escalation trial. Lancet 385(9967):517–528. 10.1016/s0140-6736(14)61403-3 25319501 10.1016/S0140-6736(14)61403-3 PMC7065359

[118] Neelapu SS, Locke FL, Bartlett NL et al (2017) Axicabtagene ciloleucel CAR T-cell therapy in refractory large B-cell lymphoma. N Engl J Med 377(26):2531–2544. 10.1056/NEJMoa1707447 29226797 10.1056/NEJMoa1707447 PMC5882485

[119] Shah D, Soper B, Shopland L (2023) Cytokine release syndrome and cancer immunotherapies - historical challenges and promising futures. Front Immunol 14:1190379. 10.3389/fimmu.2023.1190379 37304291 10.3389/fimmu.2023.1190379 PMC10248525

[120] Gust J, Ponce R, Liles WC et al (2020) Cytokines in CAR T cell-associated neurotoxicity. Front Immunol 11:577027. 10.3389/fimmu.2020.577027 33391257 10.3389/fimmu.2020.577027 PMC7772425

[121] Poh A(2017) Equipping NK Cells with CARs. Cancer Discov 7(10):of2. 10.1158/2159-8290.Cd-nb2017-124 10.1158/2159-8290.CD-NB2017-124 28877899

[122] Mehta RS, Rezvani K (2018) Chimeric antigen receptor expressing natural killer cells for the immunotherapy of cancer. Front Immunol 9:283. 10.3389/fimmu.2018.00283 29497427 10.3389/fimmu.2018.00283 PMC5818392

[123] Rezvani K, Rouce R, Liu E et al (2017) Engineering natural killer cells for cancer immunotherapy. Mol Ther 25(8):1769–1781. 10.1016/j.ymthe.2017.06.012 28668320 10.1016/j.ymthe.2017.06.012 PMC5542803

[124] Xiao L, Cen D, Gan H et al (2019) Adoptive transfer of NKG2D CAR mRNA-engineered natural killer cells in colorectal cancer patients. Mol Ther 27(6):1114–1125. 10.1016/j.ymthe.2019.03.011 30962163 10.1016/j.ymthe.2019.03.011 PMC6554529

[125] Sun C, Sun H, Zhang C et al (2015) NK cell receptor imbalance and NK cell dysfunction in HBV infection and hepatocellular carcinoma. Cell Mol Immunol 12(3):292–302. 10.1038/cmi.2014.91 25308752 10.1038/cmi.2014.91 PMC4654321

[126] Sun C, Sun HY, Xiao WH et al (2015) Natural killer cell dysfunction in hepatocellular carcinoma and NK cell-based immunotherapy. Acta Pharmacol Sin 36(10):1191–1199. 10.1038/aps.2015.41 26073325 10.1038/aps.2015.41 PMC4648180

[127] Bryceson YT, March ME, Ljunggren HG et al (2006) Synergy among receptors on resting NK cells for the activation of natural cytotoxicity and cytokine secretion. Blood 107(1):159–166. 10.1182/blood-2005-04-1351 16150947 10.1182/blood-2005-04-1351 PMC1895346

[128] Vivier E, Raulet DH, Moretta A et al (2011) Innate or adaptive immunity? The example of natural killer cells. Science 331(6013):44–49. 10.1126/science.1198687 21212348 10.1126/science.1198687 PMC3089969

[129] Ni Z, Knorr DA, Bendzick L et al (2014) Expression of chimeric receptor CD4*ζ* by natural killer cells derived from human pluripotent stem cells improves in vitro activity but does not enhance suppression of HIV infection in vivo. Stem Cells 32(4):1021–1031. 10.1002/stem.1611 24307574 10.1002/stem.1611 PMC3960346

[130] Romanski A, Uherek C, Bug G et al (2016) CD19-CAR engineered NK-92 cells are sufficient to overcome NK cell resistance in B-cell malignancies. J Cell Mol Med 20(7):1287–1294. 10.1111/jcmm.12810 27008316 10.1111/jcmm.12810 PMC4929308

[131] Zhao C, Zhang L, Zheng Z et al (2024) Case report: sudden death following the administration of CAR-NK cells for lung cancer immunotherapy. Forensic Sci Med Pathol 20(2):690–695. 10.1007/s12024-023-00693-4 37542619 10.1007/s12024-023-00693-4

[132] Liu E, Tong Y, Dotti G et al (2018) Cord blood NK cells engineered to express IL-15 and a CD19-targeted CAR show long-term persistence and potent antitumor activity. Leukemia 32(2):520–531. 10.1038/leu.2017.226 28725044 10.1038/leu.2017.226 PMC6063081

[133] Bexte T, Botezatu L, Miskey C et al (2024) Engineering of potent CAR NK cells using non-viral sleeping beauty transposition from minimalistic DNA vectors. Mol Ther 32(7):2357–2372. 10.1016/j.ymthe.2024.05.022 38751112 10.1016/j.ymthe.2024.05.022 PMC11287004

[134] Han Y, Liu D, Li L (2020) PD-1/PD-L1 pathway: current researches in cancer. Am J Cancer Res 10(3):727–742 32266087 PMC7136921

[135] Zhong Y, Liu J (2024) Emerging roles of CAR-NK cell therapies in tumor immunotherapy: current status and future directions. Cell Death Discov 10(1):318. 10.1038/s41420-024-02077-1 38987565 10.1038/s41420-024-02077-1 PMC11236993

[136] Wang W, Liu Y, He Z et al (2024) Breakthrough of solid tumor treatment: CAR-NK immunotherapy. Cell Death Discov 10(1):40. 10.1038/s41420-024-01815-9 38245520 10.1038/s41420-024-01815-9 PMC10799930

[137] Thangaraj JL, Coffey M, Lopez E et al (2024) Disruption of TGF-β signaling pathway is required to mediate effective killing of hepatocellular carcinoma by human iPSC-derived NK cells. Cell Stem Cell 31(9):1327-1343.e5. 10.1016/j.stem.2024.06.009 38986609 10.1016/j.stem.2024.06.009 PMC11380586

[138] Duan J, Zhao S, Duan Y et al (2024) Mno(x) nanoenzyme armed CAR-NK cells enhance solid tumor immunotherapy by alleviating the immunosuppressive microenvironment. Adv Healthc Mater 13(11):e2303963. 10.1002/adhm.202303963 38296248 10.1002/adhm.202303963

[139] Li Y, Basar R, Wang G et al (2024) Author correction: KIR-based inhibitory CARs overcome CAR-NK cell trogocytosis-mediated fratricide and tumor escape. Nat Med 30(3):906. 10.1038/s41591-023-02770-1 38182787 10.1038/s41591-023-02770-1

[140] Huang Y, Zeng J, Liu T et al (2020) DNAM1 and 2B4 costimulatory domains enhance the cytotoxicity of anti-GPC3 chimeric antigen receptor-modified natural killer cells against hepatocellular cancer cells in vitro. Cancer Manag Res. 10.2147/cmar.S253565 32440221 10.2147/CMAR.S253565 PMC7217313

[141] Peng L, Renauer PA, Sferruzza G et al (2024) In vivo AAV-SB-CRISPR screens of tumor-infiltrating primary NK cells identify genetic checkpoints of CAR-NK therapy. Nat Biotechnol. 10.1038/s41587-024-02282-4 38918616 10.1038/s41587-024-02282-4 PMC11668911

[142] Christodoulou I, Ho WJ, Marple A et al (2021) Engineering CAR-NK cells to secrete IL-15 sustains their anti-AML functionality but is associated with systemic toxicities. J Immunother Cancer. 10.1136/jitc-2021-003894 34896980 10.1136/jitc-2021-003894 PMC8655609

[143] Hamieh M, Dobrin A, Cabriolu A et al (2019) CAR T cell trogocytosis and cooperative killing regulate tumour antigen escape. Nature 568(7750):112–116. 10.1038/s41586-019-1054-1 30918399 10.1038/s41586-019-1054-1 PMC6707377

[144] Cho JH, Collins JJ, Wong WW (2018) Universal chimeric antigen receptors for multiplexed and logical control of T cell responses. Cell 173(6):1426-1438.e11. 10.1016/j.cell.2018.03.038 29706540 10.1016/j.cell.2018.03.038 PMC5984158

[145] Choe JH, Watchmaker PB, Simic MS et al (2021) Synnotch-CAR T cells overcome challenges of specificity, heterogeneity, and persistence in treating glioblastoma. Sci Transl Med. 10.1126/scitranslmed.abe7378 33910979 10.1126/scitranslmed.abe7378 PMC8362330

[146] Chen R, Shi X, Yao X et al (2024) Specific multivalent molecules boost CRISPR-mediated transcriptional activation. Nat Commun 15(1):7222. 10.1038/s41467-024-51694-y 39174527 10.1038/s41467-024-51694-y PMC11341856

[147] Lin JS, Lai EM (2024) Protein-Protein Interactions: Yeast Two Hybrid. Methods Mol Biol 2715:235–246. 10.1007/978-1-0716-3445-5_15 37930532 10.1007/978-1-0716-3445-5_15

[148] Martins F, Rosspopoff O, Carlevaro-Fita J et al (2024) A cluster of evolutionarily recent KRAB zinc finger proteins protects cancer cells from replicative stress-induced inflammation. Cancer Res 84(6):808–826. 10.1158/0008-5472.Can-23-1237 38345497 10.1158/0008-5472.CAN-23-1237 PMC10940857

[149] Shi Q, Xue C, Zeng Y et al (2024) Notch signaling pathway in cancer: from mechanistic insights to targeted therapies. Signal Transduct Target Ther 9(1):128. 10.1038/s41392-024-01828-x 38797752 10.1038/s41392-024-01828-x PMC11128457

[150] Hyrenius-Wittsten A, Su Y, Park M et al (2021) Synnotch CAR circuits enhance solid tumor recognition and promote persistent antitumor activity in mouse models. Sci Transl Med. 10.1126/scitranslmed.abd8836 33910981 10.1126/scitranslmed.abd8836 PMC8594452

[151] Moghimi B, Muthugounder S, Jambon S et al (2021) Preclinical assessment of the efficacy and specificity of GD2-B7H3 synnotch CAR-T in metastatic neuroblastoma. Nat Commun 12(1):511. 10.1038/s41467-020-20785-x 33479234 10.1038/s41467-020-20785-x PMC7820416

[152] Williams JZ, Allen GM, Shah D et al (2020) Precise T cell recognition programs designed by transcriptionally linking multiple receptors. Science 370(6520):1099–1104. 10.1126/science.abc6270 33243890 10.1126/science.abc6270 PMC8054651

[153] Cortese M, Torchiaro E, D’Andrea A et al (2024) Preclinical efficacy of a HER2 synNotch/CEA-CAR combinatorial immunotherapy against colorectal cancer with HER2 amplification. Mol Ther 32(8):2741–2761. 10.1016/j.ymthe.2024.06.023 38894542 10.1016/j.ymthe.2024.06.023 PMC11405179

[154] Lee YE, Go GY, Koh EY et al (2023) Synergistic therapeutic combination with a CAF inhibitor enhances CAR-NK-mediated cytotoxicity via reduction of CAF-released IL-6. J Immunother Cancer. 10.1136/jitc-2022-006130 36849201 10.1136/jitc-2022-006130 PMC9972461

[155] Huang Y, Mohanty V, Dede M et al (2023) Characterizing cancer metabolism from bulk and single-cell RNA-seq data using METAFlux. Nat Commun 14(1):4883. 10.1038/s41467-023-40457-w 37573313 10.1038/s41467-023-40457-w PMC10423258

[156] Hosseinalizadeh H, Wang LS, Mirzaei H et al (2025) Emerging combined CAR-NK cell therapies in cancer treatment: finding a dancing partner. Mol Ther. 10.1016/j.ymthe.2024.12.057 39754357 10.1016/j.ymthe.2024.12.057 PMC12172187

[157] Li J, Hu H, Lian K et al (2024) CAR-NK cells in combination therapy against cancer: a potential paradigm. Heliyon 10(5):e27196. 10.1016/j.heliyon.2024.e27196 38486782 10.1016/j.heliyon.2024.e27196 PMC10937699

[158] Lupo KB, Yao X, Borde S et al (2024) Synnotch-programmed iPSC-derived NK cells usurp TIGIT and CD73 activities for glioblastoma therapy. Nat Commun 15(1):1909. 10.1038/s41467-024-46343-3 38429294 10.1038/s41467-024-46343-3 PMC10907695

[159] Ahmadnia A, Mohammadi S, Yamchi A et al (2024) Augmenting the antitumor efficacy of natural killer cells via synnotch receptor engineering for targeted IL-12 secretion. Curr Issues Mol Biol 46(4):2931–2945. 10.3390/cimb46040183 38666913 10.3390/cimb46040183 PMC11048765

[160] Ma M, Badeti S, Chen CH et al (2021) CAR-NK cells effectively target the D614 and G614 SARS-CoV-2 infected cells. BioRxiv. 10.1101/2021.01.14.42674234981061

[161] Mazarzaei A, Vafaei M, Ghasemian A et al (2019) Memory and CAR-NK cell-based novel approaches for HIV vaccination and eradication. J Cell Physiol 234(9):14812–14817. 10.1002/jcp.28280 30779120 10.1002/jcp.28280

