# Supplementary material for: Precision sniper for solid tumors: CAR-NK cell therapy
Source: Cancer Immunol Immunother. 2025 Jul 24;74(9):275. doi: 10.1007/s00262-025-04106-z (PMC12290171; doi:10.1007/s00262-025-04106-z)
Supplement: Supplementary file 1 — Supplementary file1 (DOCX 666 kb) [file 262_2025_4106_MOESM1_ESM.docx]

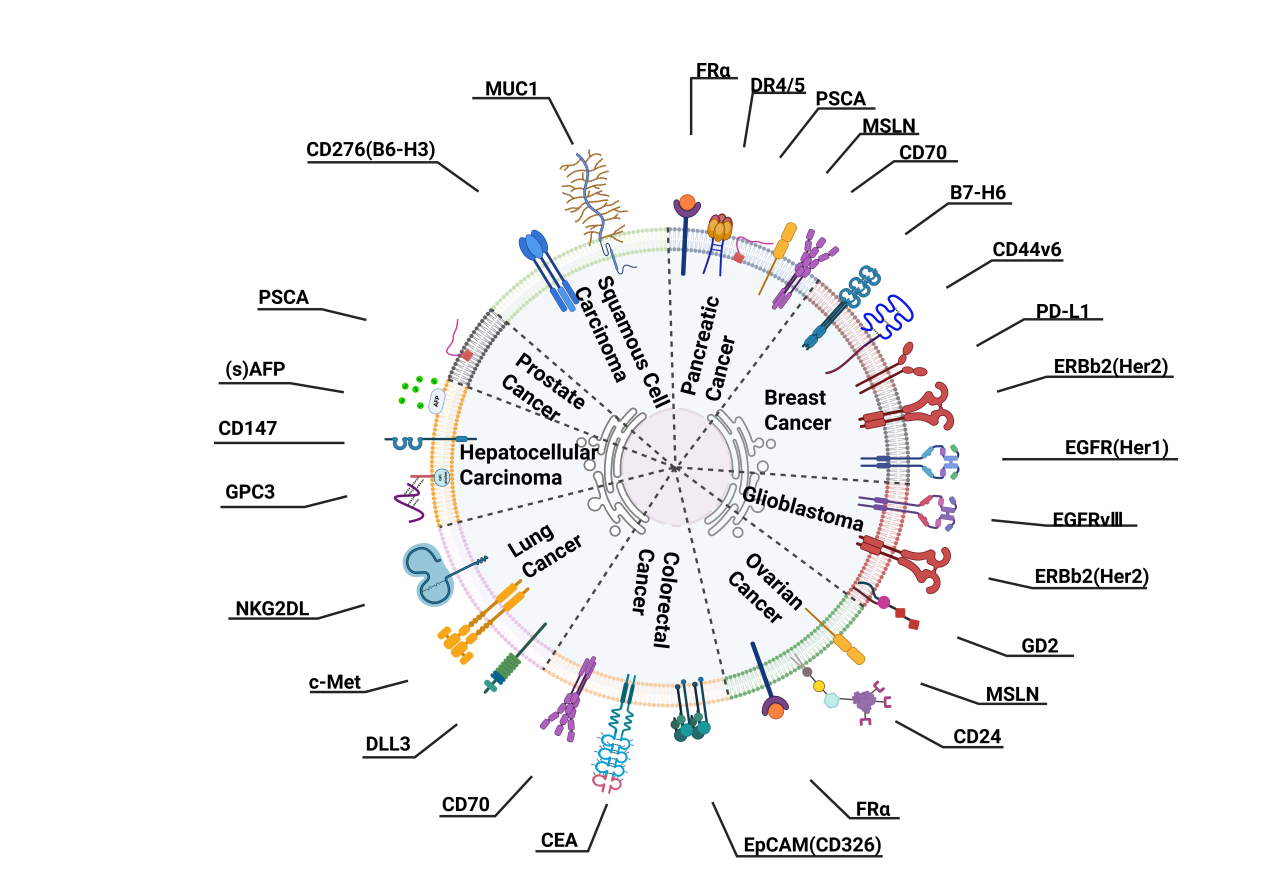


Fig. 1 Summary of CAR-NK cell treatment targets for different solid tumors. This figure was drawn via Biorender


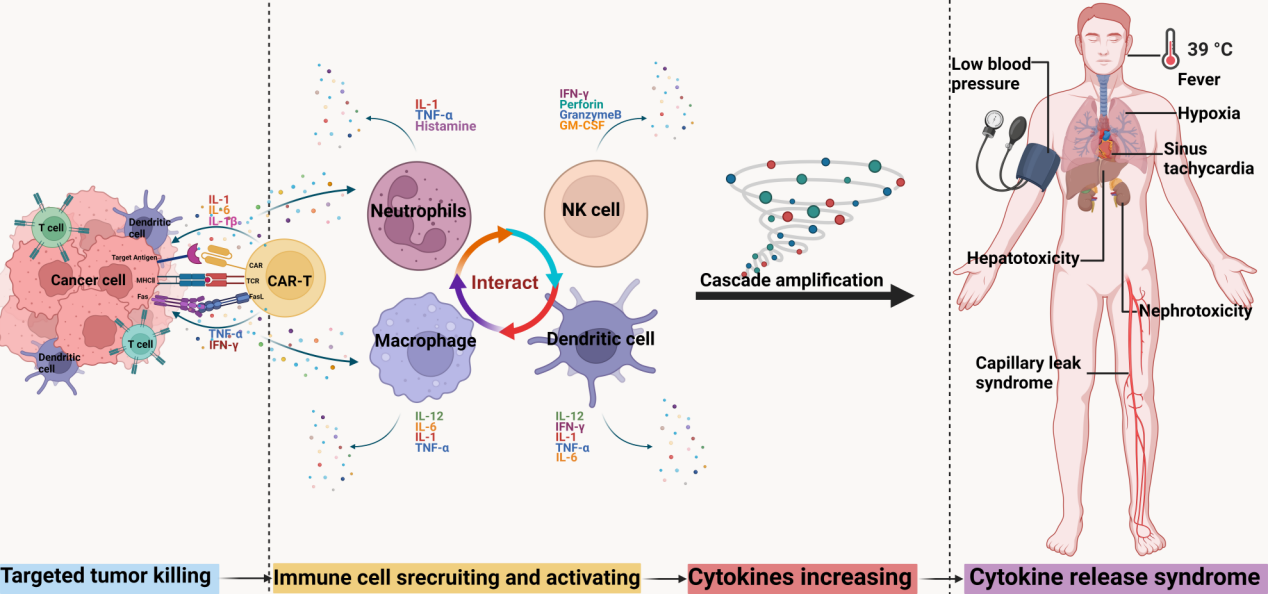


Fig. 2 CRS caused by CAR-T-cell therapy. CAR-T-cell-secreted cytokines that kill tumor cells activate neutrophils, NK cells, macrophages, and DCs and induce them to release more inflammatory cytokines, causing cascade amplification reactions that cause CRS. Clinical symptoms include fever, low blood pressure, hypoxia, sinus tachycardia, hepatotoxicity, nephrotoxicity and capillary leakage syndrome. This figure was drawn via Biorender
